# The dynamics of* bla*_TEM_ resistance genes in *Salmonella* Typhi

**DOI:** 10.1038/s41598-024-74321-8

**Published:** 2024-10-16

**Authors:** Narong Nuanmuang, Pimlapas Leekitcharoenphon, Patrick Murigu Kamau Njage, Alix Vincent Thorn, Frank M. Aarestrup

**Affiliations:** Research Group for Genomic Epidemiology, National Food Institute, Technical University of Denmark, Kgs. Lyngby, Denmark

**Keywords:** *Salmonella* Typhi, Dynamics, Antimicrobial resistance, Antibiotic resistance gene, Flanking region pattern, Computational biology and bioinformatics, Genetics, Microbiology

## Abstract

*Salmonella* Typhi (*S*. Typhi) is an important pathogen causing typhoid fever worldwide. The emergence of antibiotic resistance, including that of *bla*_TEM_ genes encoding to TEM $\:\beta\:$-lactamases has been observed. This study aimed to investigate the dynamics of *bla*_TEM_ genes in *S*. Typhi by analyzing the phylogeny and flanking region patterns and phylogenetic associating them with metadata (year, country) and genomic data (genotypes, antibiotic resistance genes (ARGs), plasmids). Genomic sequences of publicly available *S*. Typhi harboring *bla*_TEM_ (n = 6079), spanning from 1983 to 2023, were downloaded and analyzed using CSIPhylogeny for phylogeny, Flankophile for identifying genetic contexts around *bla*_TEM_ genes and GenoTyphi for determining genotypes, ARGs and plasmid replicons. We found that *bla*_TEM_-positive isolates occurred most commonly in specific location, especially in Asia and Africa and clustered among a limited number of genotypes. Flankophile identified 740 isolates (12.2%) with distinct flanking region patterns, which were categorized into 13 patterns. Notably, 7 patterns showed a predominantly phylogenetic association with genotypes. Additionally, these 7 patterns exhibited relation to the country, ARGs and plasmid replicons. Further examination of the flanking region patterns provided association with mobile genetic elements (MGEs). Taken together, this study suggests that *bla*_TEM_ has been acquired by *S*. Typhi isolates a limited number of times and subsequently spread clonally with specific genotypes.

## Introduction

*Salmonella* Typhi (*S*. Typhi) remains an important pathogen causing typhoid fever^1^. It causes approximately 11 million cases and 110,000 deaths globally each year^2,3^ is particularly a public health concern in low- and middle-income countries^2,4–8^, but also in high-income countries remaining relevant due to travel associated infections^1,8–11^.

Antimicrobial resistance (AMR) poses a major threat to human and animal health^12^. Studies have reported a widespread emergence in *S*. Typhi isolates^5,6,9,13,14^. Among AMR mechanisms in *S.* Typhi, the production of TEM *β*-lactamase, expressed from *bla*_TEM_ gene, plays a crucial role in conferring resistance to *β*-lactam drugs^9,15,16^. The *bla*_TEM_ gene family encompasses more than 201 variants^17^. Among these, an important variant reported in *S*. Typhi is *bla*_TEM-1B_^18^.

Antibiotic resistance genes (ARGs) can be transferred on a large range of mobile genetic elements (MGEs), including plasmids, integrons (Int), insertion sequences (IS) and transposons (Tn)^15^. There is, however, limited knowledge on the dynamics of *bla*_TEM_ in *S*. Typhi populations. To investigate this, we obtained genomic sequences along with sample metadata from publicly available online sources. Our primary focus was on exploring the dynamics of *bla*_TEM_ by examining flanking region patterns, the sequences surrounding the targeted resistance gene. We then explored phylogenetic association between these patterns, sample metadata and analyzed genomic data.

## Materials and methods

### Selection of ***S***. Typhi and ***bla***_TEM_ harboring isolates

We gathered *S*. Typhi isolates from three different databases: Pathogenwatch^19^ (n = 13,041), Enterobase^20^ (n = 11,548), the NCBI Pathogen Detection project^21^ (n = 11,688). The metadata from these databases were accessed on 27^th^ November 2023 and combined. Duplicate accession numbers and non-Typhi isolates were excluded and a total of 19,354 unique *S*. Typhi were retrieved for the subsequent selection of *bla*_TEM_ isolates. Based on the metadata provided these isolates were categorized into: (1) non-*bla*_TEM_ harboring *S*. Typhi (n = 13,275) and (2) *S*. Typhi harboring *bla*_TEM_ isolates (n = 6079) that were used for studying of dynamics of *bla*_TEM_.

### Genomic sequence downloading and analysis

The genomic data for the 6079 selected *S*. Typhi harboring *bla*_TEM_ isolates, were downloaded from ENA Portal API. The sequences underwent processing, including pre- and post-trimming quality checks, as well as assembly, using the FoodQCPipeline^22^.

Briefly, raw reads were examined for quality using FastQC v0.11.5 (Babraham Bioinformatics, 2016), followed by trimming of sequencing adaptors using bbduk2 (part of BBtools v36.49, https://jgi.doe.gov/data-and-tools/software-tools/bbtools/) with an internal database. Subsequently, the trimmed raw reads were re-evaluated for quality using FastQC. All reads that passed quality were according to: (1) read length was longer or equal to 50 base pairs (bp), (2) phred score per base was higher or equal to 20 and (3) adaptors were removed. The quality-passed reads were then de novo assembled using SPAdes v3.11.0 (Bankevich, et. al., 2012). The resulting assemblies were evaluated for quality using Quast v 4.5 (https://gitlab.univ-lille.fr/mathieu.genete/ngsgenotyp/-/tree/master/TOOLS/quast-4.5).

The confirmation of serovars was conducted using SISTR^23^. The *bla*_TEM_ variants were confirmed using ABricate v1.0.1 (https://github.com/tseemann/abricate) with three databases, AMRFinderPlus^24^, ResFinder^25^ and CARD^26^. Genotypes, ARG profiles and plasmid replicon profiles were determined using Genotyphi v2.0^27^. The distribution of the genotypes was visualized using Microreact (https://microreact.org/project/6aHVsxbEyr1WKsFjqM9wgC-styphiblatemv1)^28^.

### Phylogenetic tree construction

The assembled genomes of the 6079 *S*. Typhi harboring *bla*_TEM_ were aligned to the reference genome of *S*. Typhi CT18, an XDR strain from Vietnam in the year 1993 (accession no: AL513382) using CSIPhylogeny pipeline^29^. The resulting core SNP alignment was used for inferring a maximum-likelihood tree with Fastree v2.1.3^30^. The phylogenetic tree (SNP Tree), sample metadata and analyzed genomic results were then visualized by Microreact^28^.

### Generating* bla*_*TEM*_ gene synteny

The *bla*_TEM_ gene synteny was analyzed using Flankophile v0.2.7, a tool for analyzing and visualization of genetic context surrounding the targeted resistance gene (gene synteny). The contigs carrying *bla*_TEM_ of the 6079 selected *S*. Typhi isolates were used as an input. Concerning Flankophile configurations: (1) ResFinder (08/02/2022) database was utilized with a minimum coverage and identity to the reference sequence of > 98%, (2) flanking length (both upstream and downstream) was defined as 2000 base pairs (bp), (3) cluster identity and length differences were set at 0.98, (4) k-mer size indexing was set at 16 and (5) distance matrix calculation was conducted using method number one which involves k-mer hamming distance^31^. The Flankophile outputs were analyzed and reported as the flanking region patterns.

## Results

### Temporal and geographical distribution of *Salmonella* Typhi isolates

**Fig. 1:**
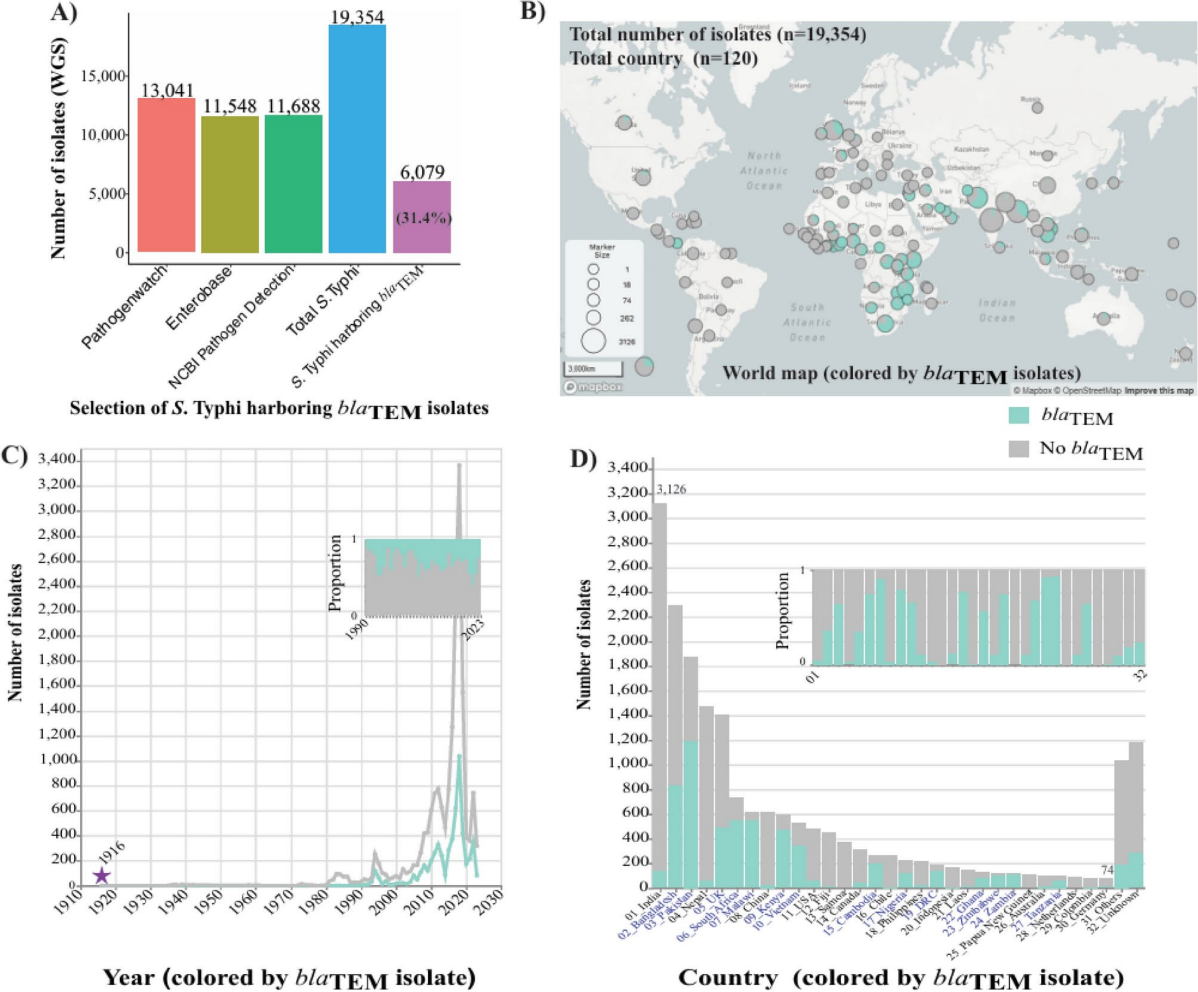
Acquisition and distribution of *Salmonella* Typhi isolates (harboring vs non-harboring *bla*_TEM_). Acquisition of *S.* Typhi harboring *bla*_TEM_ isolates from publicly available online database (**A**). The distribution of *S*. Typhi isolates (harboring vs non-harboring *bla*_TEM_) shows as the world map (**B**), year of isolation **(C**) and country of origin (**D**). Legend, green is *S.* Typhi harboring *bla*_TEM_ isolates (n = 6079) and grey is non-harboring *bla*_TEM_ isolates (n = 13,275).

All *bla*_TEM_ harboring genomic sequences were downloaded, re-confirmed serovar Typhi using SISTR and classified as *S*. Typhi harboring *bla*_TEM_ isolates (n = 6079; 31.4%). While isolates that did not harbor *bla*_TEM_ were classified as non-*bla*_TEM_ harboring *S*. Typhi (n = 13,275; 68.6%) (Fig. 1 & Supplementary Table 1). Most *bla*_TEM_ variant were *bla*_TEM-1B_ (n = 6037), but we also found *bla*_TEM-135_ (n = 8), *bla*_TEM-116_ (n = 1), *bla*_TEM-215_ (n = 1) and *bla*_TEM-others_ (n = 32). Among all *bla*_TEM_-positive isolates, there were 851 isolates showing co-existence with ESBL genes. One of the most common ESBL genes was *bla*_CTX-M-15_*β*-lactamase gene (n = 834/851) (Supplementary Table 2).

Our dataset revealed information of *S*. Typhi isolates, spanning from 1916 to 2023 with the first *bla*_TEM_-positive isolates observed from Chile in 1983 (Supplementary Table 3). Although the number of *bla*_TEM_ harboring isolates has been increasing based on sequencing input, the proportion of *bla*_TEM_-positive isolates remained relatively stable over time. The *bla*_TEM_-positive isolates were predominantly from Asia (Southern, South-eastern) and Africa (Eastern, Western and Southern) and had a more geographically restricted distribution than *bla*_TEM_-negative isolates (Fig. 1).

### Association of *bla*_TEM_ to genotypes

**Fig. 2:**
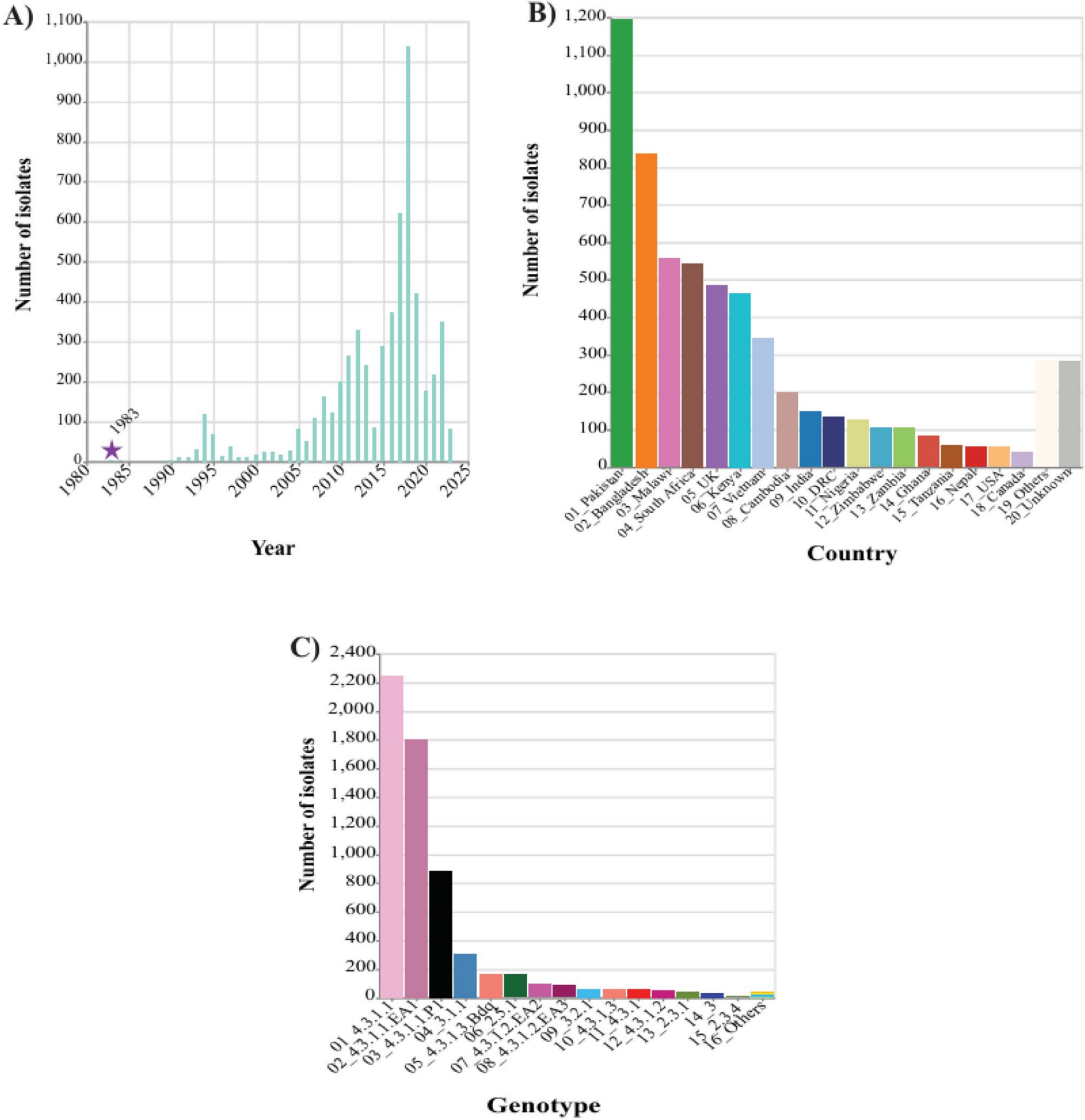
Distribution of *Salmonella* Typhi harboring *bla*_TEM_ isolates. The distribution of the selected isolates (n = 6079) is divided by year (**A**), country (**B**) and genotype (**C**).

The distribution of *bla*_TEM_-positive *S*. Typhi isolates was also compared to genotypes. The *bla*_TEM_-negative isolates exhibited greater genotypic diversity of genotype than the *bla*_TEM_-positive isolates and the later isolates were mainly found in genotype 4.3.1.1, 4.3.1.1.EA1, 4.3.1.1.P1, 3.1.1, 4.3.1.3.Bdq and and 2.5.1, mainly from Asia and Africa (Fig. 2 and Supplementary Fig. 1 & Supplementary Table 4).

### Associations between phylogeny, genotypes, plasmid and resistance profiles of *bla*_TEM_ positive isolates

The 6079 *S*. Typhi harboring *bla*_TEM_ isolates were used for constructing maximum-likelihood phylogenetic tree. SNP-analyses showed large genomic diversity among the *bla*_TEM_-positive isolates, with the phylogenetic tree in general clustering with the defined genotypes (Supplementary Fig. 2). However, some discrepancies were also observed. Thus, especially genotype 4.3.1.1 clustered in groups into different parts of the phylogeny, genotypes 2.3.1, 2.3.4, 2.5.1, 3, 3.1.1 and 3.2.1 closely together, as did genotypes 4.3.1, 4.3.1.2, 4.3.1.2.EA2, 4.3.2.2.EA3, 4.3.1.3 and 4.3.1.3.Bdq. There was also some clustering of isolates from the same countries, but in several cases, clones were observed across several countries and even continents. The antibiotic resistance gene (ARG) profile also showed association to phylogeny. Almost all *bla*_TEM_-positive isolates also harbored *catA1*, *dfrA*7, *sul1* and *sul2*. However, exceptions were observed (Supplementary Fig. 2).

A limited number of isolates harbored *bla*_CTX-M-15_, but even though this was identified in two different resistance profiles and across the different phylogenetic clusters, no clear association to plasmids was observed. One exception was a very clear association between resistance profile 10 (negative for *catA1*, *tet*(B) and *sul2*) and plasmid profile 6 (IncFIB(K) among isolates from Bangladesh (Supplementary Fig. 2). All ARG profiles were classified in to 16 profiles (Supplementary Fig. 3 and Supplementary Table 5). All plasmid profiles were classified in to 13 plasmid profiles (Supplementary Table 6).

### Analysis of *bla*_TEM_ flanking region patterns

**Fig. 3:**
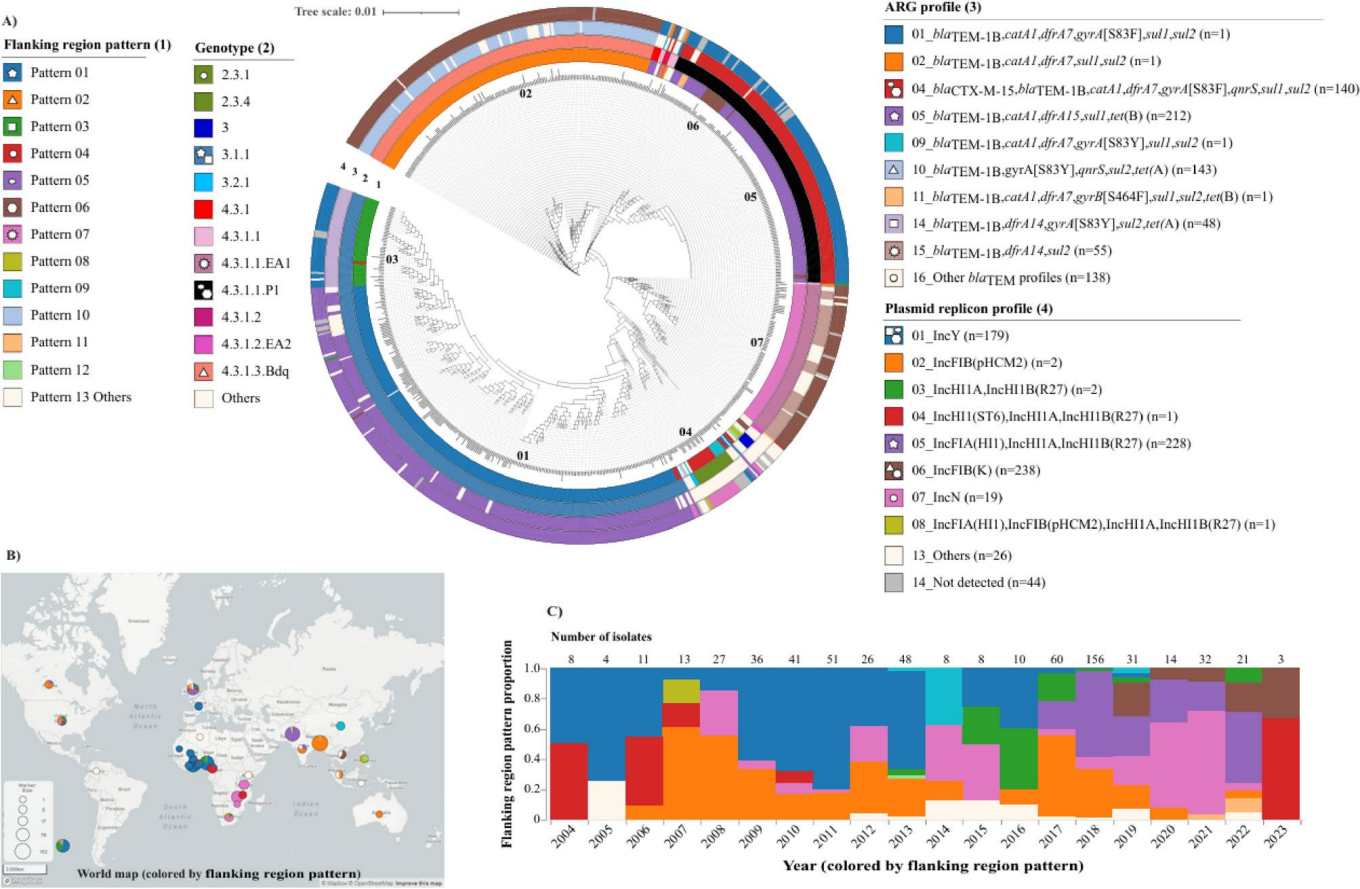
Association of flanking region patterns with analyzed genomic data. The flanking region patterns (1) are linked to genotype (2), antibiotic resistance gene (ARG) profile (3) and plasmid replicon profile (4) (**A**). The *S*. Typhi harboring *bla*_TEM_ isolates with flankophile outputs (n = 740) are used for constructing a SNP Tree using CT18 as reference strain. The tree has 36,350 SNP and 76.5% coverage. The distribution of flanking region pattern of *S*. Typhi harboring *bla*_TEM_ isolates shows as the world map (**B**). The dynamics of flanking region patterns is shown between 2004 and 2023 (**C**). The symbols of seven common flanking region patterns, pattern 01 (five point star), pattern 02 (triangle), pattern 03 (square), pattern 04 (circle), pattern 05 (oval), pattern 06 (hexagon) and pattern 07 (multipoint star), depict linkage of the flanking region pattern with other metadata.

It was only possible to obtain sufficient flanking regions (2000 bp on each side) for 740 isolates (12.2%) (Supplementary Fig. 4 and Supplementary Table 7). Those isolates were not equally distributed across the phylogenetic tree, but were mainly from genotype 3.1.1 in Nigeria and Ghana, 4.3.1.3.Bdq in Bangladesh, 4.3.1.1.P1 in Pakistan and 4.3.1.1.EA1 in Zambia and Tanzania (Fig. 3 and Supplementary Figs. 5 and 6). Flanks of 5000 bp were available from 577 isolates (Supplementary Fig. 7 and Supplementary Table 8).

The flankophile outputs were then categorized into 13 different patterns (01–13). Among them, there were 7 dominant patterns, namely pattern 01 (n = 240), pattern 02 (n = 167), pattern 03 (n = 48), pattern 04 (n = 22), pattern 05 (n = 128), pattern 06 (n = 19) and pattern 07 (n = 80) (Supplementary Fig. 4 and Supplementary Table 7).

The seven dominant flanking patterns (01–07) were closely linked to genotype, ARG profile and plasmid profile, but less to country of origin and isolation year (Fig. 3 and Supplementary Figs. 5 and 6).

Flanking region pattern (Pattern) 01 clustered with genotype 3.1.1-mainly isolated from Nigeria and Ghana, plasmid profile 5 and ARG profile 5. However, Pattern 03 clustered phylogenetically here and with a different plasmid profile namely IncY (profile 1) and ARG profile 14. Pattern 03 was only linked with genotype 3.1.1 and was mainly isolated from Nigeria. However, in 3 isolates the pattern was linked to USA (n = 2) and UK (n = 1) (Fig. 3 and Supplementary Figs. 5 and 6).

Pattern 02 was associated with 4.3.1.3.Bdq, mainly originated from Bangladesh and IncFIB(K) (plasmid profile 6) and ARG profile 10. Pattern 04 was observed among 22 isolates and distributed quite broadly across the phylogeny, but associated to plasmid profile 7 (IncN) (Fig. 3 and Supplementary Figs. 5 and 6).

Pattern 05 clustered with genotype 4.3.1.1.P1, was mainly isolated from Pakistan and associated with ARG profile 4 and plasmid pattern 1 (IncY). Pattern 06 was also related to genotype 4.3.1.1.P1, ARG profile 4 and plasmid profile 1 and was observed from various countries, Pakistan (n = 5), UK (n = 5), USA (n = 3). Pattern 07 clustered with 4.3.1.1.EA1, was isolated from Africa (Zambia and Tanzania), mainly associated to ARG profile 15 and plasmid profile 6 (IncFIB(K) (Fig. 3 and Supplementary Figs. 5 and 6).

**Fig. 4:**
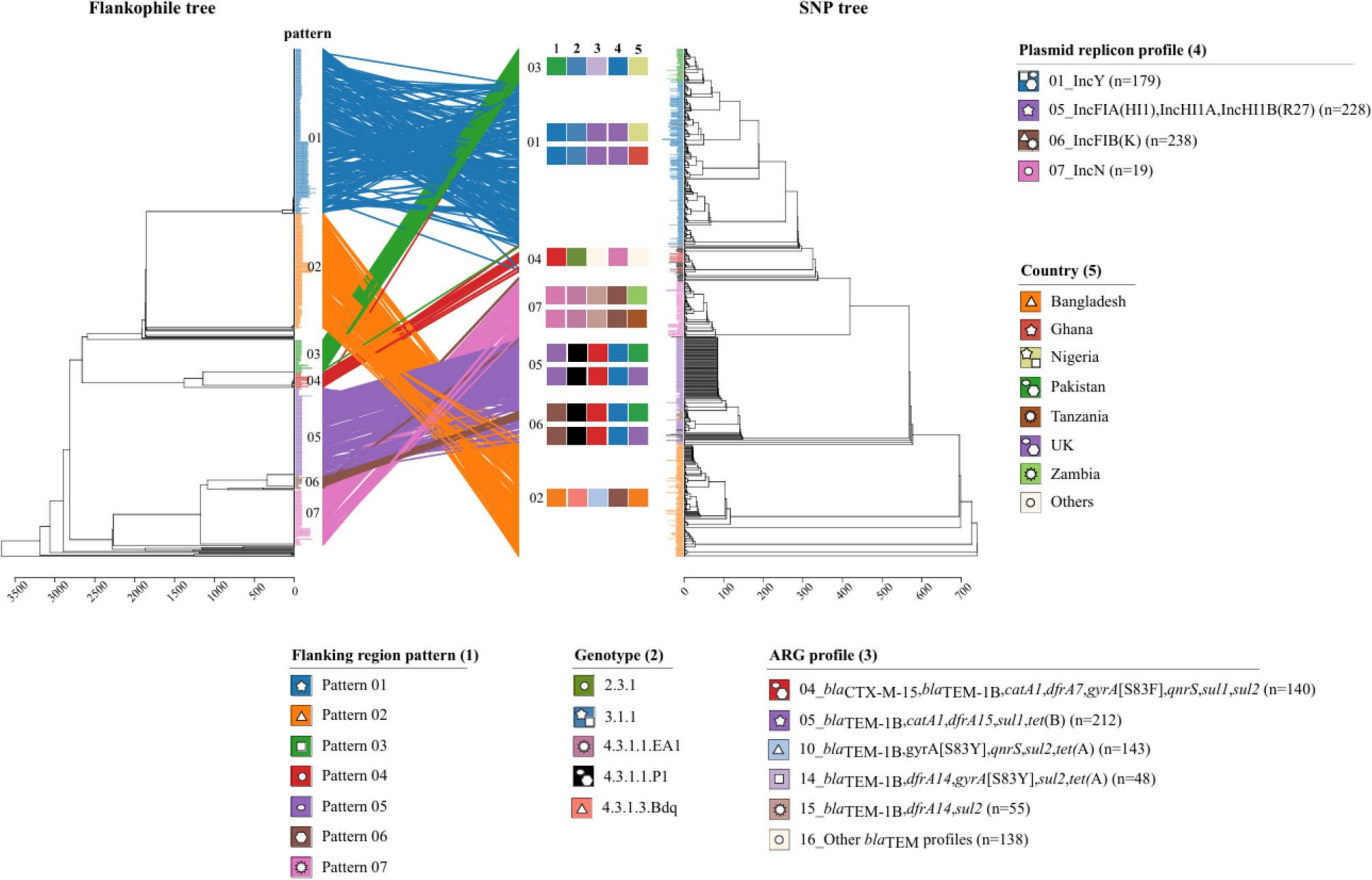
Clustering of flanking region patterns of* S*. Typhi harboring *bla*_TEM_ isolates. The clustering is linked between a flankophile tree constructed using Flankophile and a SNP tree constructed using CSIPhylogeny. The clustering represents association between flanking region pattern (1) and genotype (2), ARG profile (3), plasmid replicon profile (4) and country (5). The symbols of seven common flanking region patterns, pattern 01 (five point star), pattern 02 (triangle), pattern 03 (square), pattern 04 (circle), pattern 05 (oval), pattern 06 (hexagon) and pattern 07 (multipoint star), depict linkage of the flanking region pattern with other metadata.

The major clustering of SNP-phylogeny and flanking region pattern is also confirmed and illustrated using tanglegram (Fig. 4).

We further analyzed the genetic contexts surrounding *bla*_TEM_, specifically focusing on insertion sequences, within a 5000 bp flanking region using the Flankophile tool. Pattern 01 was included upstream Tn2 (n = 240). Pattern 02 was contained upstream Tn2 and downstream ISVsa3 and IS5075 (n = 160). Pattern 03 was contained upstream ISAs25 and ISEc63 (n = 47). Pattern 04 was included upstream Tn2 (n = 20). Pattern 05 was comprised upstream ISEcp1 and downstream IS5075 (n = 11). Pattern 07 was upstream ISEc63 and downstream IS5075 (n = 75). Overall, Tn2 emerged as the most common upstream element, while IS5075 was identified as the dominant downstream element (Supplementary Fig. 7 and Supplementary Table 8).

All flanking region patterns were illustrated individually for clustering with metadata profile (genotype, ARG profile, plasmid profile and country) (Supplementary Figs. 8–20).

## Discussion

### ***S***. Typhi harboring*** bla***_TEM_

Our study focused on *S*. Typhi harboring *bla*_TEM_ isolates obtained from publicly available data. Major geographical differences in *S*. Typhi harboring *bla*_TEM_ were found and a high prevalence of the *bla*_TEM_ isolates was dominantly observed in Asia and Africa. Despite the presence of surveillance programs, the number of reported cases remains underestimated, particularly in developing countries. Enhanced surveillance and accurate reporting are essential for determining the true distribution of resistance and for collecting data to inform public health decisions and policies. The *bla*_TEM-1B_ was the most common variant found in the *S*. Typhi harboring *bla*_TEM_ isolates (99.3%), and only a few other variants (*bla*_TEM-135_, *bla*_TEM-215_) were observed. The co-existence of *bla*_TEM-1B_ and *bla*_CTX-M-15_ (n = 834), encoding resistance to cephalosporins, was predominantly observed (13.7%). This result is consistent with several previous reports on high prevalence of *bla*_TEM_ genes in these regions^2,32^. The isolates carrying the *bla*_CTX-M-15_ gene exhibited XDR properties^5^.

Genotyping is a valuable approach for examining local *S*. Typhi populations and detecting recent introductions of specific genotypes in new or re-endemic areas^33,34^. *S*. Typhi harboring *bla*_TEM_ isolates were occurred in a limit number of genotypes, especially genotypes 4.3.1.1, 4.3.1.1.P1, 4.3.1.3.Bdq, 4.3.1.1.EA1, 3.1.1 and 2.5.1. While non-*bla*_TEM_ isolates varied much more in genotypes and geographical isolation location.

In general, a strong correlation between genotypes, ARG profile and plasmid profiles were observed, suggesting that *bla*_TEM_ have not emerged multiple times among several clones of *S*. Typhi, but more likely that acquisition has only occurred a few times followed by subsequent spread of resistant variants. *S.* Typhi harboring *bla*_TEM_ mainly belonged to genotypes 4.3.1 and its sublineages. This result is consistent with a report by Silva et al., which estimated that the first transfer of 4.3.1 (H58) originated from India and subsequently spread internationally and intercontinentally^35^. Genotype 4.3.1 (H58) has recently disseminated across different continents^34^. It was commonly reported in Asia (Bangladesh, Pakistan, Nepal and India)^6,7^, Africa (Kenya, East, West)^10,36,37^ and individuals returning from endemic countries, including the UK and Australia^1,9^.

### Dynamics of flanking region patterns

We also performed an analysis of flanking regions of the *bla*_TEM_ genes. This could only be done for a subset of the data due to the unequal quality of the sequencing data. However, we found some common MGEs, Tn2, ISEc63, ISEcp1 and IS5075. This suggests that *bla*_TEM_, surrounded by mobile genetic elements (MGEs), particularly Tn2, has the potential for transmission and altering the genes surrounding the *bla*_TEM_ gene. The flanking region analyses in general confirmed that *bla*_TEM_ has been acquired a limited number of times followed by subsequent spread of the new resistant clones. However, a few cases of possible horizontal transmission were also found.

This study is the first largest analysis of flanking regions of *bla*_TEM_-positive isolates and flanking region analyses thus supports the initial findings, but we also observed that a better quality of data is needed to perform such analyses.

### Limitations and suggestions

The sample metadata lacking information on serovars or ARGs was excluded, potentially leading to an underestimation of the total number of eligible isolates. In the flankophile output, only 740 isolates (12.2%) successfully passed the default setting with a flanking length of 2000 bp. Exclusions were based on contig length and the percentage of identity and coverage (% < 98). Therefore, enhancing sequencing quality by obtaining longer contigs or utilizing sequences from long-read platforms could significantly improve the genomic information available for studying the evolution and dynamics of targeted resistance genes using the Flankophile tool.

## Conclusion

Our analysis of publicly available *S*. Typhi genomes showed *bla*_TEM_ harboring isolates were most commonly found in Asia and Africa and distributed in a limit number of genotypes, especially 4.3.1 and its sublineages. The *bla*_TEM_ flanking region patterns were dominantly associated to genotypes and associated with ARG profiles, plasmid replicons and countries. Our study suggests that *bla*_TEM_ has been independently acquired by a limited number of *S*. Typhi genotypes and likely with subsequent spread of the resistant variants regionally. However, while clonal transmission of resistant variants seems to be the most important reason for *bla*_TEM_ emergence, a few cases of horizontal transmission between genotypes was also observed.

## Supplementary Information

Supplementary Information.

## Data Availability

All metadata used in this study can be searched at: https://microreact.org/project/6aHVsxbEyr1WKsFjqM9wgC-styphiblatemv1.
