# Supplementary material for: The dynamics of blaTEM resistance genes in Salmonella Typhi
Source: Sci Rep. 2024 Oct 16;14:24311. doi: 10.1038/s41598-024-74321-8 (PMC11484844; doi:10.1038/s41598-024-74321-8)
Supplement: Supplementary file 1 — Supplementary Information. [file 41598_2024_74321_MOESM1_ESM.pdf]

Supplementary Material

Supplementary tables and figures

Supplementary table 1. The number of *Salmonella* Typhi isolates divided by countries. The total number of *S*. Typhi isolates divided by countries (n=19,354) (1.1), *S*. Typhi harboring *bla*$_{TEM}$ isolates divided by countries (n=6,079) (1.2) and non-*bla*$_{TEM}$ harboring *S*. Typhi divided by countries (n=13,275) (1.3).

1.1)

Country	No of isolate
01_India	3126
02_Bangladesh	2296
03_Pakistan	1876
04_Nepal	1468
05_UK	1401
06_South Africa	727
07_Malawi	615
08_China	610
09_Kenya	590
10_Vietnam	527
11_USA	471
12_Fiji	448
13_Samoa	365
14_Canada	310
15_Cambodia	262
16_Chile	258
17_Nigeria	223
18_Philippines	214
19_DRC	179
20_Indonesia	163
21_Laos	142
22_Ghana	122
23_Zimbabwe	114
24_Zambia	110
25_Papua New Guinea	105
26_Australia	96
27_Tanzania	93
28_Netherlands	84
29_Colombia	76
30_Germany	74
31_Others	1033
32_Unknown	1176
Total	19354

1.2)

Country by *bla* $_{TEM}$ isolate	No of isolate
01_Pakistan	1193
02_Bangladesh	837
03_Malawi	556
04_South Africa	543
05_UK	485
06_Kenya	464
07_Vietnam	344
08_Cambodia	200
09_India	146
10_DRC	132
11_Nigeria	125
12_Zimbabwe	104
13_Zambia	103
14_Ghana	82
15_Tanzania	59
16_Nepal	54
17_USA	52
18_Canada	39
19_Others	281
20_Unknown	280
Total	6079

Supplementary table 1. The number of *Salmonella* Typhi isolates divided by countries. The total number of *S*. Typhi isolates divided by countries (n=19,354) (1.1), *S*. Typhi harboring bla_{TEM} isolates divided by countries (n=6,079) (1.2) and non-bla_{TEM} harboring *S*. Typhi divided by countries (n=13,275) (1.3).

1.3)

**Country by non-bla_{TEM} isolate**	**No of isolate**
01_India	2980
02_Bangladesh	1459
03_Nepal	1414
04_UK	916
05_Pakistan	683
06_China	590
07_Fiji	433
08_USA	419
09_Samoa	365
10_Canada	271
11_Chile	257
12_Philippines	191
13_South Africa	184
14_Vietnam	183
15_Indonesia	161
16_Laos	127
17_Kenya	126
18_Papua New Guinea	105
19_Nigeria	98
20_Australia	85
21_Netherlands	84
22_Colombia	75
23_Germany	67
24_Cambodia	62
25_Malawi	59
26_DRC	47
27_Ghana	40
28_Tanzania	34
29_Zimbabwe	10
30_Zambia	7
31_Others	847
32_Unknown	896
Total	13275

Supplementary table 2. The number of *Salmonella* Typhi harboring bla_{TEM} and ESBL gene isolates. The number of the isolates (n=851) (2.1) classified by ESBL variant (2.2), year (2.3), country of origin (2.4), genotype (2.5), ARG profile (2.6), plasmid profile (2.7) and flanking region pattern (2.8).

2.1)

bla $_{TEM}$ **isolate by ESBL**	**No of isolate**
ESBL	851
non ESBL	5200
Unknown	28
Total	6079

2.2)

bla $_{TEM}$**_ESBL isolate**	**No of isolate**
$bla_{CTX-M-15}$,bla_{TEM-1B}	834
$bla_{TEM-135}$	8
$bla_{CTX-M-15}$,bla_{TEM}	4
bla_{SHV-12},bla_{TEM-1B}	2
$bla_{TEM-215}$	1
$bla_{CTX-M-12}$,bla_{TEM-1B}	1
$bla_{CTX-M-55}$,bla_{TEM-1B}	1
Total	851

2.3)

bla $_{TEM}$**_ESBL isolate by year**	**No of isolate**
2000	2
2007	3
2008	1
2010	2
2012	1
2013	2
2014	1
2015	3
2016	18
2017	129
2018	407
2019	94
2020	51
2021	37
2022	90
2023	5
Unknown	5
Total	851

2.4)

bla $_{TEM}$**_ESBL isolate by country of origin**	**No of isolate**
Pakistan	672
UK	118
USA	14
Oman	10
Unknown	9
Philippines	5
India	4
Australia	3
South Africa	3
Bangladesh	2
Indonesia	2
Malawi	2
DRC	1
Mexico	1
China	1
Cameroon	1
Colombia	1
Canada	1
Sri Lanka	1
Total	851

Supplementary table 2. The number of *Salmonella* Typhi harboring bla_{TEM} and ESBL gene isolates. The number of the isolates (n=851) (2.1) classified by ESBL variant (2.2), year (2.3), country of origin (2.4), genotype (2.5), ARG profile (2.6), plasmid profile (2.7) and flanking region pattern (2.8).

2.5)

***bla* TEM_ESBL isolate by genotype**	**No of isolate**
4.3.1.1.P1	825
4.3.1	7
3	5
1	2
4.3.1.1	2
2.4.1	2
3.3.2	2
4.3.1.1.EA1	1
4.3.1.2	1
2.5.1	1
2.3.1	1
3.5	1
3.2.1	1
Total	851

2.6)

***bla* TEM_ESBL isolate by ARG profile**	**No of isolate**
04_ $bla_{CTX-M-15}$, bla_{TEM-1B}, *catA1*, *dfrA7*, *gyrA*[S83F], *qnrS*, *sul1*, *sul2*	487
07_ $bla_{CTX-M-15}$, bla_{TEM-1B}, *catA1*, *dfrA7*, *gyrA*[S83F], *qnrS*, *sul1*	155
11_ $bla_{CTX-M-15}$, bla_{TEM-1B}, *catA1*, *dfrA7*, *gyrA*[S83F], *qnrS1*, *sul1*, *sul2*	117
16_Other bla_{TEM} profiles	92
Total	851

2.7)

***bla* TEM_ESBL isolate by plasmid profile**	**No of isolate**
01_IncY	700
07_IncN	7
22_Others	7
23_Not detected	137
Total	851

2.8)

***bla* TEM_ESBL isolate by flanking region pattern**	**No of isolate**
Pattern 04	3
Pattern 05	128
Pattern 06	16
Pattern 08	2
Pattern 10	2
Pattern 11	3
Pattern 13 Others	3
Pattern 14 No output	694
Total	851

Supplementary table 3. The number of *Salmonella* Typhi isolates divided by years. The total number of *S*. Typhi isolates divided by years (n=19,354) (3.1), *S*. Typhi harboring bla_{TEM} isolates divided by years (n=6,079) (3.2) and non-bla_{TEM} harboring *S*. Typhi divided by years (n=13,275) (3.3).

3.1)

Year	No of isolate
1916	1
1935	1
1936	5
1937	9
1938	1
1939	10
1941	4
1942	5
1958	4
1960	3
1961	2
1972	2
1973	13
1976	3
1977	1
1980	4
1981	36
1982	49
1983	94
1984	91
1985	73
1986	73
1987	29
1988	28
1989	27
1990	33
1991	52
1992	45
1993	62
1994	252
1995	179
1996	104
1997	89
1998	52
1999	64
2000	78
2001	62
2002	132
2003	101
2004	115
2005	165
2006	165
2007	264
2008	418
2009	427
2010	603
2011	739
2012	775
2013	626
2014	464
2015	772
2016	1271
2017	2391
2018	3367
2019	1549
2020	382
2021	360
2022	744
2023	319
Unknown	1565
Total	19354

3.2)

Year by *bla* $_{TEM}$ isolate	No of isolate
1983 (Chile)	1
1984	1
1990	4
1991	8
1992	9
1993	30
1994	119
1995	66
1996	12
1997	37
1998	8
1999	8
2000	16
2001	23
2002	23
2003	15
2004	28
2005	82
2006	51
2007	108
2008	163
2009	121
2010	201
2011	263
2012	329
2013	240
2014	85
2015	287
2016	373
2017	621
2018	1037
2019	419
2020	175
2021	217
2022	347
2023	82
Unknown	470
Total	6079

Supplementary table 3. The number of *Salmonella* Typhi isolates divided by years. The total number of *S*. Typhi isolates divided by years (n=19,354) (3.1), *S*. Typhi harboring bla_{TEM} isolates divided by years (n=6,079) (3.2) and non-bla_{TEM} harboring *S*. Typhi divided by years (n=13,275) (3.3).

3.3)

Year by non-bla_{TEM} isolate	No of isolate
1916	1
1935	1
1936	5
1937	9
1938	1
1939	10
1941	4
1942	5
1958	4
1960	3
1961	2
1972	2
1973	13
1976	3
1977	1
1980	4
1981	36
1982	49
1983	93
1984	90
1985	73
1986	73
1987	29
1988	28
1989	27
1990	29
1991	44
1992	36
1993	32
1994	133
1995	113
1996	92
1997	52
1998	44
1999	56
2000	62
2001	39
2002	109
2003	86
2004	87
2005	83
2006	114
2007	156
2008	255
2009	306
2010	402
2011	476
2012	446
2013	386
2014	379
2015	485
2016	898
2017	1770
2018	2330
2019	1130
2020	207
2021	143
2022	397
2023	237
Unknown	1095
Total	13275

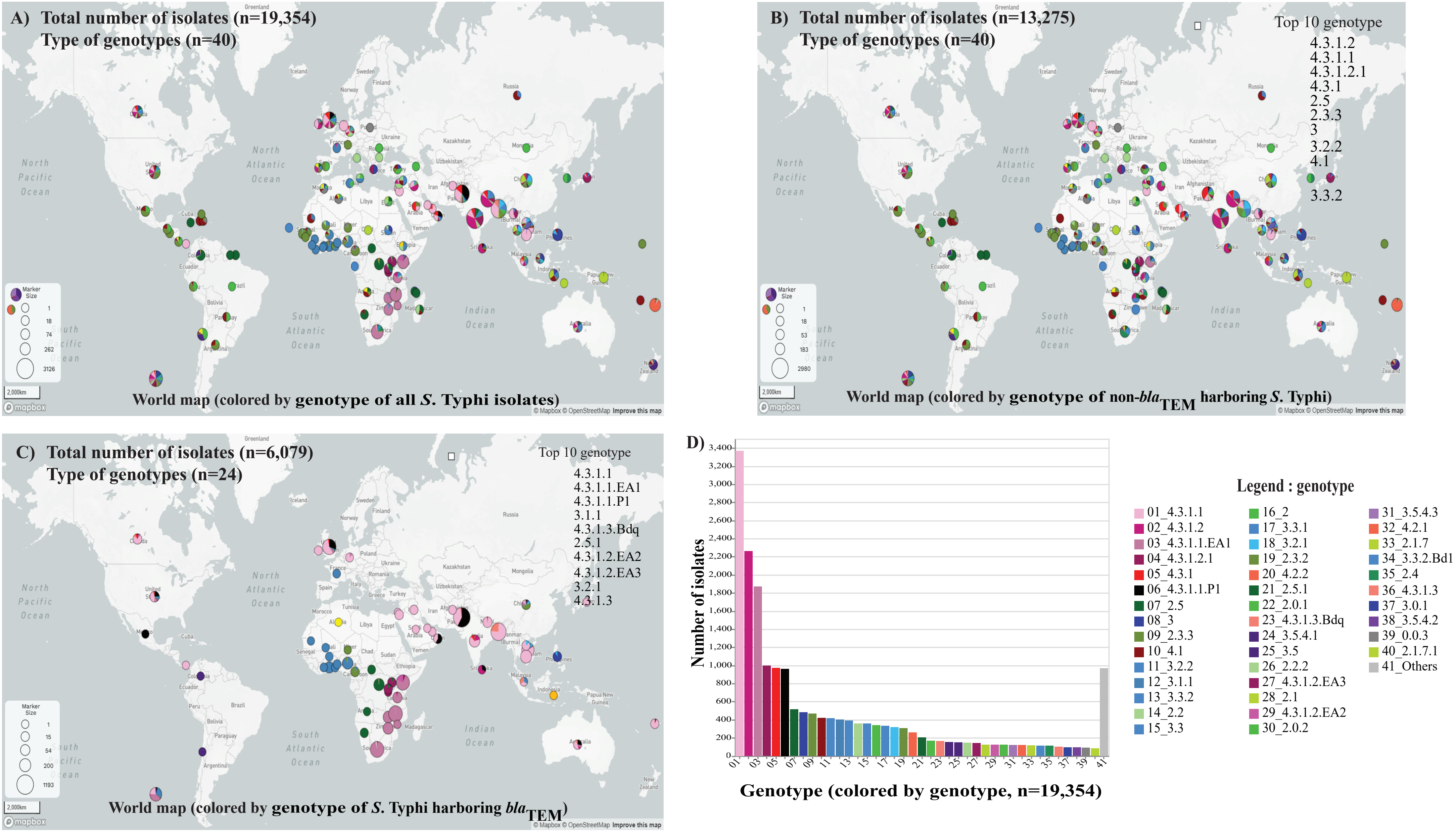

Supplementary figure 1. Distribution of *Salmonella* Typhi harboring *bla*TEM isolates classified by genotypes. The genotypic distribution shows the total number of *S.* Typhi isolates (n=19,354) (**A, D**), Non-*bla*TEM harboring *S.* Typhi isolates (n=13,275) (**B**) and *S.* Typhi harboring *bla*TEM isolates (6,079) (**C**).

Supplementary table 4. The number of *Salmonella* Typhi isolates divided by genotypes. The total number of *S.* Typhi isolates divided by genotypes (n=19,354) (4.1), *S.* Typhi harboring bla_{TEM} isolates divided by genotypes (n=6,079) (4.2) and non- bla_{TEM} harboring *S.* Typhi divided by genotypes (n=13,275) (4.3).

4.1)

Genotype	No of isolate
01_4.3.1.1	3371
02_4.3.1.2	2261
03_4.3.1.1.EA1	1871
04_4.3.1.2.1	997
05_4.3.1	973
06_4.3.1.1.P1	963
07_2.5	515
08_3	482
09_2.3.3	470
10_4.1	421
11_3.2.2	419
12_3.1.1	402
13_3.3.2	393
14_2.2	359
15_3.3	358
16_2	339
17_3.3.1	334
18_3.2.1	319
19_2.3.2	309
20_4.2.2	261
21_2.5.1	204
22_2.0.1	168
23_4.3.1.3.Bdq	167
24_3.5.4.1	156
25_3.5	152
26_2.2.2	147
27_4.3.1.2.EA3	144
28_2.1	126
29_4.3.1.2.EA2	124
30_2.0.2	124
31_3.5.4.3	122
32_4.2.1	120
33_2.1.7	116
34_3.3.2.Bd1	115
35_2.4	113
36_4.3.1.3	103
37_3.0.1	96
38_3.5.4.2	94
39_0.0.3	93
40_2.1.7.1	84
41_Others	969
Total	19354

4.2)

Genotype by *bla* $_{TEM}$ isolate	No of isolate
01_4.3.1.1	2244
02_4.3.1.1.EA1	1799
03_4.3.1.1.P1	883
04_3.1.1	307
05_4.3.1.3.Bdq	166
06_2.5.1	158
07_4.3.1.2.EA2	92
08_4.3.1.2.EA3	84
09_3.2.1	60
10_4.3.1.3	59
11_4.3.1	58
12_4.3.1.2	51
13_2.3.1	41
14_3	30
15_2.3.4	9
16_Others	38
Total	6079

Supplementary table 4. The number of *Salmonella* Typhi isolates divided by genotypes. The total number of *S*. Typhi isolates divided by genotypes (n=19,354) (4.1), *S*. Typhi harboring bla_{TEM} isolates divided by genotypes (n=6,079) (4.2) and non- bla_{TEM} harboring *S*. Typhi divided by genotypes (n=13,275) (4.3).

4.3)

Genotype by non-bla_{TEM} isolate	No of isolate
01_4.3.1.2	2210
02_4.3.1.1	1127
03_4.3.1.2.1	997
04_4.3.1	915
05_2.5	514
06_2.3.3	469
07_3	452
08_3.2.2	418
09_4.1	413
10_3.3.2	390
11_3.3	358
12_2.2	357
13_2	339
14_3.3.1	334
15_2.3.2	309
16_4.2.2	261
17_3.2.1	259
18_2.0.1	168
19_3.5.4.1	156
20_3.5	150
21_2.2.2	145
22_2.1	126
23_2.0.2	124
24_3.5.4.3	122
25_4.2.1	120
26_2.1.7	116
27_3.3.2.Bd1	115
28_2.4	110
29_3.0.1	95
30_3.1.1	95
31_3.5.4.2	94
32_0.0.3	92
33_2.1.7.1	84
34_4.3.1.1.P1	80
35_4.3.1.1.EA1	72
36_4.3.1.2.EA3	60
37_2.5.1	46
38_4.3.1.3	44
39_4.3.1.2.EA2	32
40_4.3.1.3.Bdq	1
41_Others	906
Total	13275

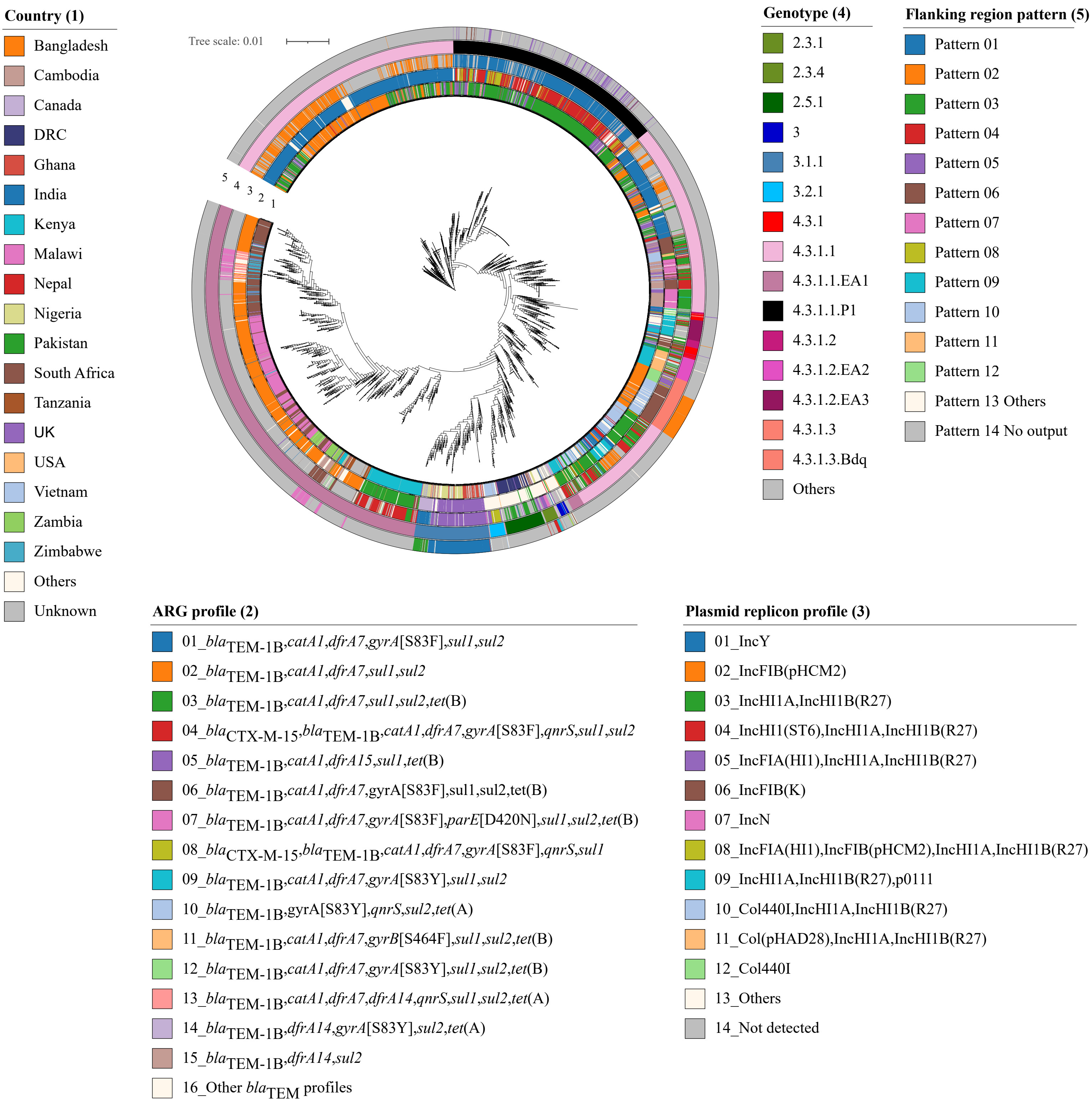

Supplementary figure 2. Distribution of flanking region patterns among *Salmonella* Typhi harboring bla_{TEM} isolates. The phylogenetic tree (SNP tree) of *S.* Typhi harboring bla_{TEM} is linked to country (1), ARG profile (2), plasmid replicon profile (3), genotype (4) and flanking region pattern (5). The tree was constructed from SNP differences of housekeeping genes of selected isolates (n=6,079).

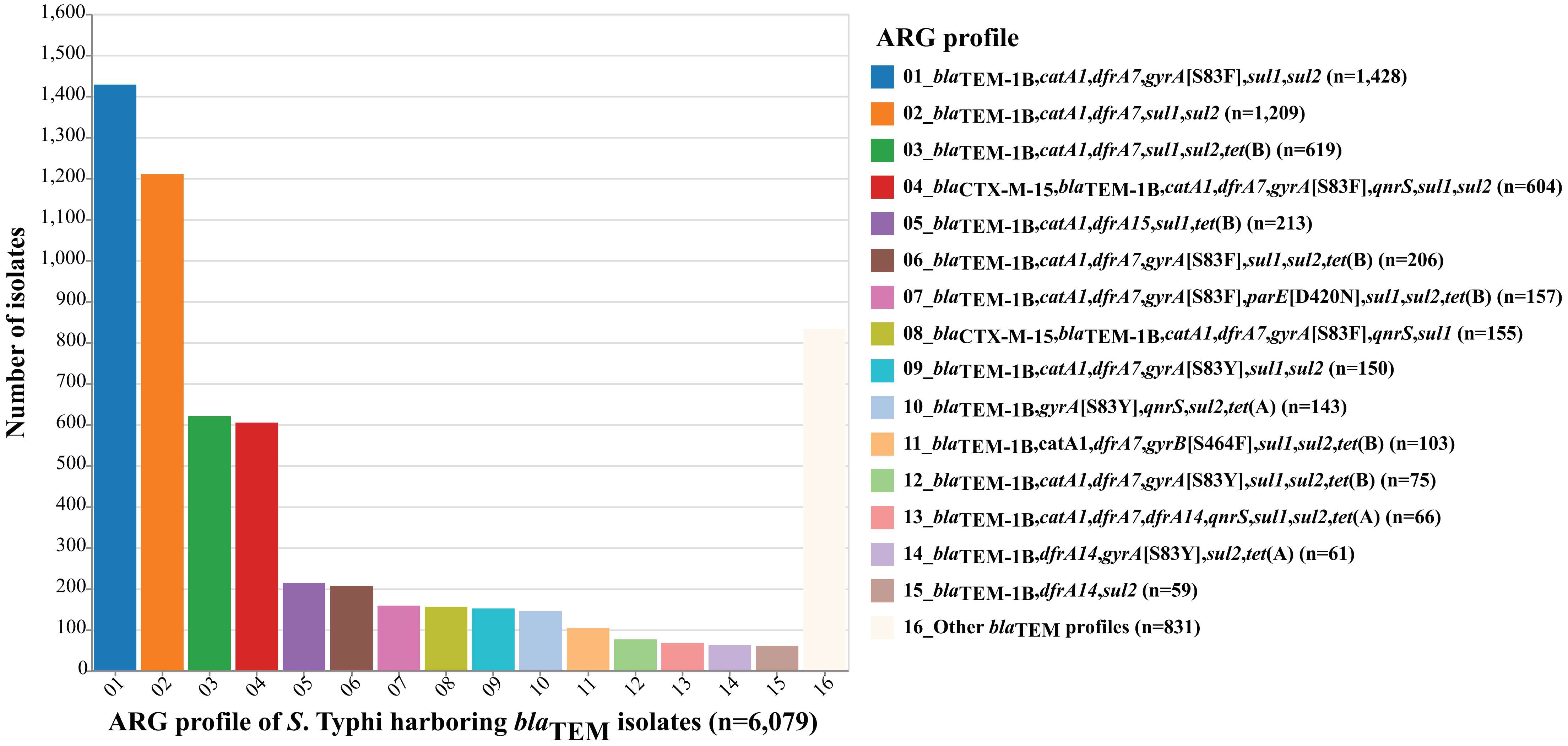

Supplementary figure 3. Antibiotic resistance gene profiles classified in this study. Profile 01-16 belonged to *Salmonella* Typhi harboring *bla*$_{TEM-1B}$ isolates with different antibiotic resistance genes.

Supplementary table 5. Antibiotic resistance gene profiles classified in this study. Profile 01-16 belonged to *Salmonella* Typhi harboring $bla_{TEM\text{-}1B}$ isolates.

bla **TEM isolate by ARG profile**	**No of isolate**
01_*bla*$_{TEM\text{-}1B}$,*catA1*,*dfrA7*,*gyrA*[S83F],*sul1*,*sul2*	1428
02_*bla*$_{TEM\text{-}1B}$,*catA1*,*dfrA7*,*sul1*,*sul2*	1209
03_*bla*$_{TEM\text{-}1B}$,*catA1*,*dfrA7*,*sul1*,*sul2*,*tet(*B)	619
04_*bla*$_{CTX\text{-}M\text{-}15}$,*bla*$_{TEM\text{-}1B}$,*catA1*,*dfrA7*,*gyrA*[S83F],*qnrS*,*sul1*,*sul2*	604
05_*bla*$_{TEM\text{-}1B}$,*catA1*,*dfrA15*,*sul1*,*tet*(B)	213
06_*bla*$_{TEM\text{-}1B}$,*catA1*,*dfrA7*,*gyrA*[S83F],*sul1*,*sul2*,*tet*(B)	206
07_*bla*$_{TEM\text{-}1B}$,*catA1*,*dfrA7*,*gyrA*[S83F],*parE*[D420N],*sul1*,*sul2*,*tet*(B)	157
08_*bla*$_{CTX\text{-}M\text{-}15}$,*bla*$_{TEM\text{-}1B}$,*catA1*,*dfrA7*,*gyrA*[S83F],*qnrS*,*sul1*	155
09_*bla*$_{TEM\text{-}1B}$,*catA1*,*dfrA7*,*gyrA*[S83Y],*sul1*,*sul2*	150
10_*bla*$_{TEM\text{-}1B}$,*gyrA*[S83Y],*qnrS*,*sul2*,*tet*(A)	143
11_*bla*$_{TEM\text{-}1B}$,*catA1*,*dfrA7*,*gyrB*[S464F],*sul1*,*sul2*,*tet*(B)	103
12_*bla*$_{TEM\text{-}1B}$,*catA1*,*dfrA7*,*gyrA*[S83Y],*sul1*,*sul2*,*tet*(B)	75
13_*bla*$_{TEM\text{-}1B}$,*catA1*,*dfrA7*,*dfrA14*,*qnrS*,*sul1*,*sul2*,*tet*(A)	66
14_*bla*$_{TEM\text{-}1B}$,*dfrA14*,*gyrA*[S83Y],*sul2*,*tet*(A)	61
15_*bla*$_{TEM\text{-}1B}$,*dfrA14*,*sul2*	59
16_Other *bla*$_{TEM}$ profile	831
Total	6079

Supplementary table 6. Plasmid replicon profiles classified in this study. Profile 01-13 belonged to *Salmonella* Typhi harboring *bla*$_{TEM\text{-}1B}$ isolates.

bla $_{\mathbf{TEM}}$ **isolate by plasmid replicon profile**	**No of isolate**
01_IncY	762
02_IncFIB(pHCM2)	722
03_IncHI1A,IncHI1B(R27)	530
04_IncHI1(ST6),IncHI1A,IncHI1B(R27)	497
05_IncFIA(HI1),IncHI1A,IncHI1B(R27)	345
06_IncFIB(K)	247
07_IncN	141
08_IncFIAHI1,IncFIB(pHCM2),IncHI1A,IncHI1B(R27)	34
09_IncHI1A,IncHI1B(R27),p0111	29
10_Col440I,IncHI1A,IncHI1B(R27)	24
11_Col(pHAD28),IncHI1A,IncHI1B(R27)	11
12_Col440I	11
13_Others	131
14_Not detected	2595
Total	6079

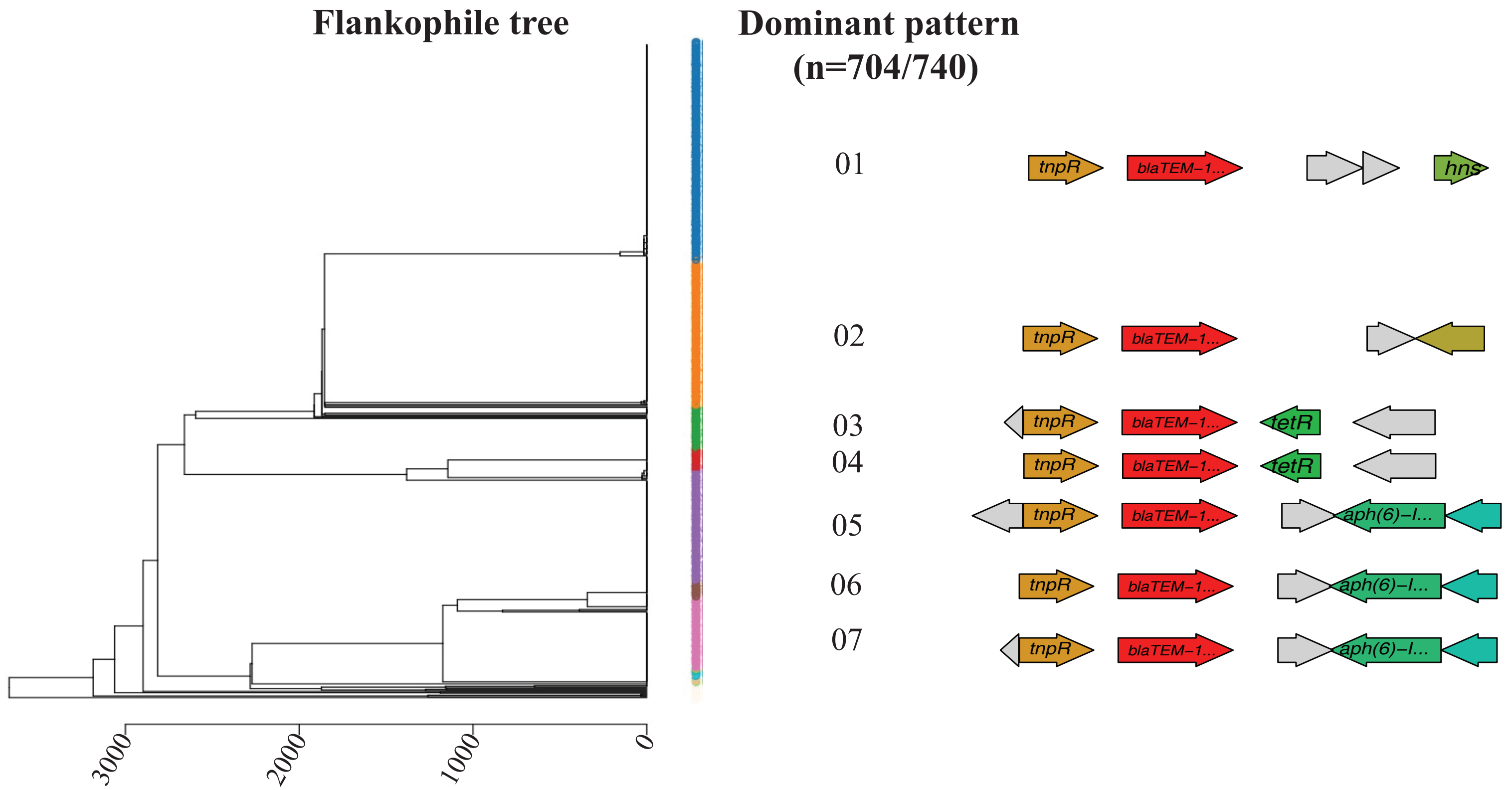

Legend : Flanking region pattern (2,000 bp flanking region, n=740)

- **Pattern 01** ***tnpR*,*bla*$_{\textit{TEM-1B}}$,hp,hp,*hns* (n=240)**
- **Pattern 02** ***tnpR*,*bla*$_{\textit{TEM-1B}}$,hp,*sul2* (n=167)**
- **Pattern 03** **hp,*tnpR*,*bla*$_{\textit{TEM-1B}}$,*tetR*,hp (n=48)**
- **Pattern 04** ***tnpR*,*bla*$_{\textit{TEM-1B}}$,*tetR*,hp (n=22)***
- **Pattern 05** **hp,*tnpR*,*bla*$_{\textit{TEM-1B}}$,hp,*aph(6)-Id*,*aph(3'')-Ib* (n=128)**
- **Pattern 06** ***tnpR*,*bla*$_{\textit{TEM-1B}}$,hp,*aph(6)-Id*,*aph(3'')-Ib* (n=19)**
- **Pattern 07** **hp,*tnpR*,*bla*$_{\textit{TEM-1B}}$,hp,*aph(6)-Id*,*aph(3'')-Ib* (n=80)**
- **Pattern 08** ***tnpR*,*bla*$_{\textit{TEM-1B}}$,hp (n=2)**
- **Pattern 09** ***tnpR*,*bla*$_{\textit{TEM-1B}}$,hp (n=7)**
- **Pattern 10** **hp,*tnpR*,*bla*$_{\textit{TEM}}$,hp (n=2)**
- **Pattern 11** **hp,*tnpR*,*bla*$_{\textit{TEM-1B}}$,hp,IS26 (n=3)**
- **Pattern 12** **hp,*tnpR*,*bla*$_{\textit{TEM-1B}}$,hp (n=3)**
- **Pattern 13** **Others (n=19)**

Supplementary figure 4. Flanking region patterns classified in this study. The pattern 01-13 are classified from flankophile outputs (n=740) with flanking region, 2,000 bp. Seven dominant flanking region patterns, 01-07 (n=704/740; 95%), represent as gene orientation. The symbol * represents the Pattern 04 including other *bla*$_{\text{TEM}}$ variants such as *bla*$_{\text{TEM-135}}$ (n=2) and *bla*$_{\text{TEM-215}}$ (n=1). The grey arrowed symbol is hypothetical genes used for pattern classification.

Supplementary table 7. Flanking region patterns classified in this study. Pattern 01-13 belonged to *Salmonella* Typhi harboring $bla_{\text{TEM-1B}}$ isolates.

bla **TEM isolate by flanking region pattern**	**No of isolate**
Pattern 01	240
Pattern 02	167
Pattern 03	48
Pattern 04	22
Pattern 05	128
Pattern 06	19
Pattern 07	80
Pattern 08	2
Pattern 09	7
Pattern 10	2
Pattern 11	3
Pattern 12	3
Pattern 13 Others	19
Pattern 14 No output	5339
Total	6079

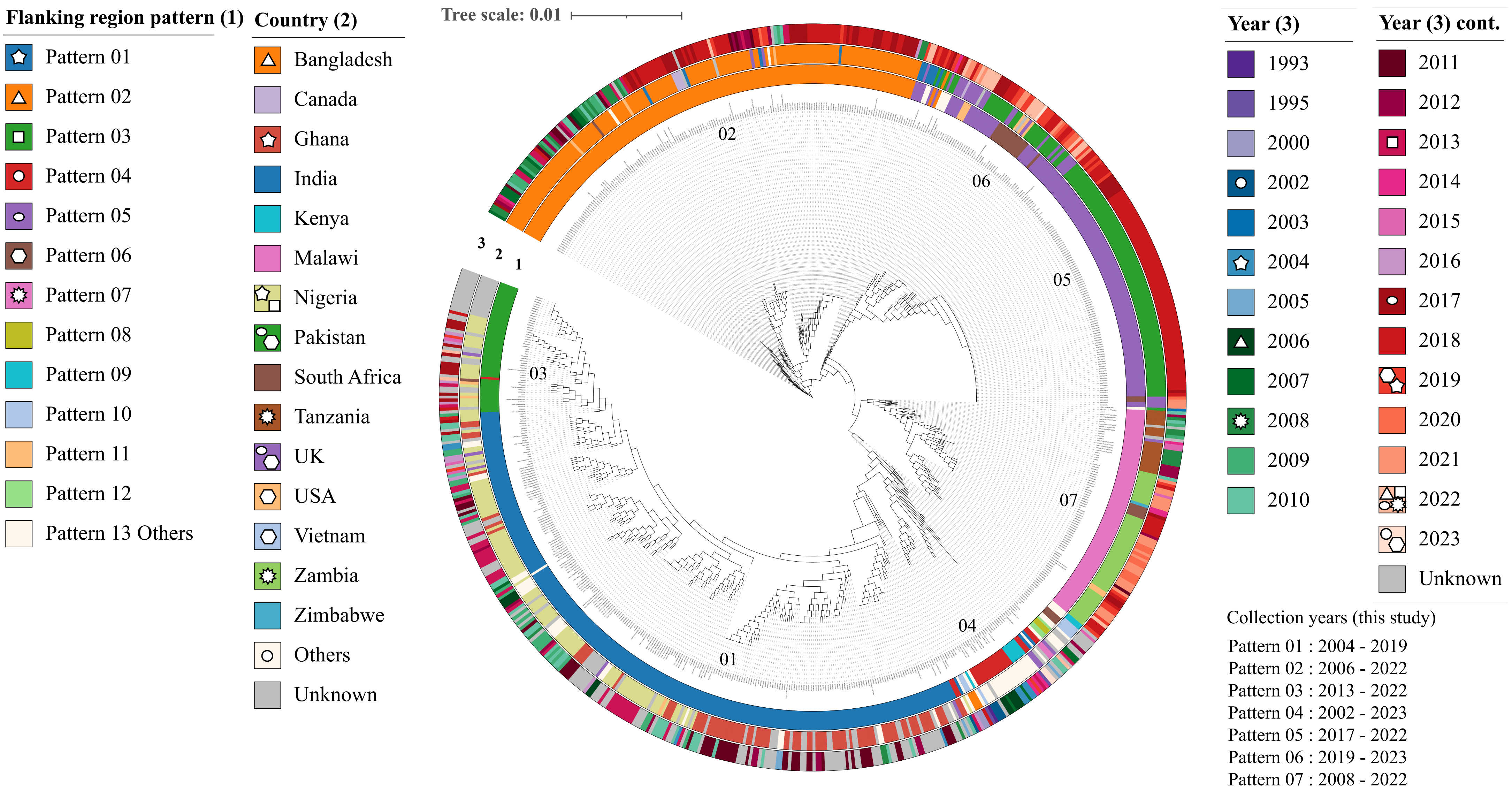

Supplementary figure 5. Association of flanking region patterns with analyzed genomic data. The flanking region patterns (1) are linked to country (2) and year (3). The tree was constructed from SNP differences of housekeeping genes of *S*. Typhi harboring bla_{TEM} isolates with flankophile output (n=740) using CT18 as reference strain.

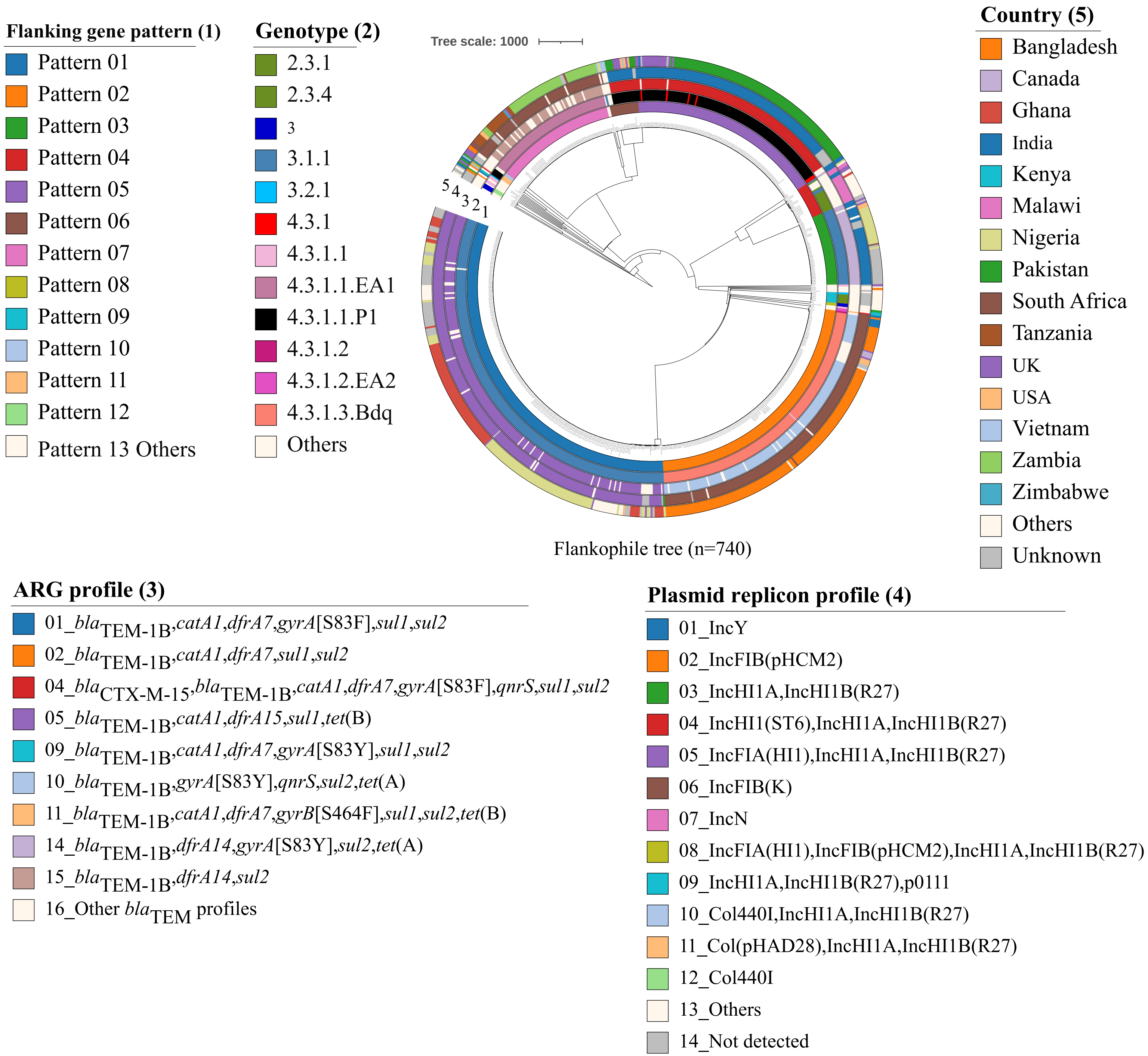

Supplementary figure 6. Association of flanking region patterns with analyzed genomic data. The flanking region patterns (1) are linked to genotype (2), ARG profile (3), plasmid replicon profile (4) and country (5). The tree is made from distance matrices based on hierarchical clustering of *bla*$_{TEM}$ and *bla*$_{TEM}$ flanking regions of *S.* Typhi harboring *bla*$_{TEM}$ isolates with flankophile output (n=740).

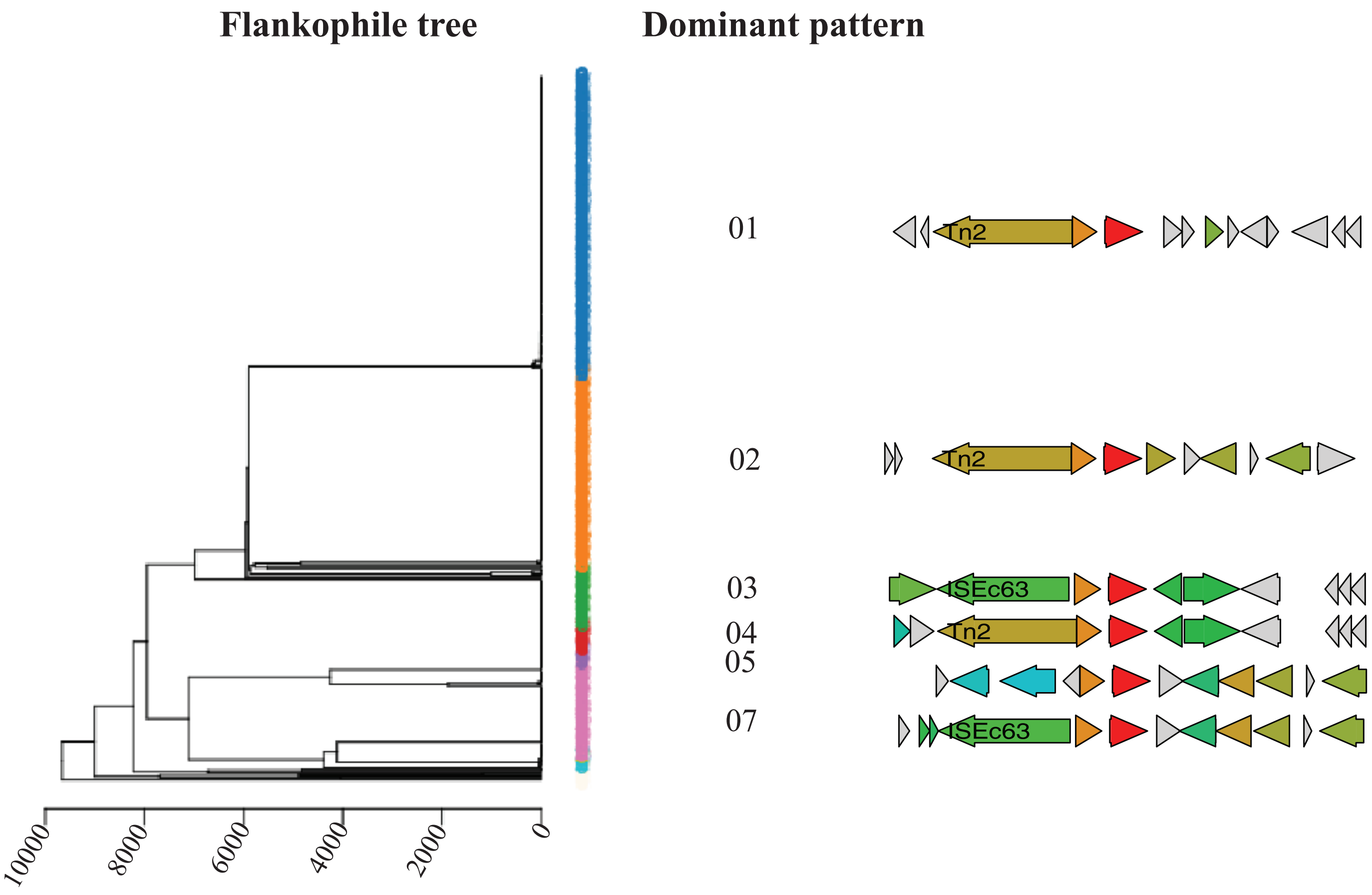

Legend : Dominant flanking region pattern (5,000 bp flanking region, n=553/577)

Pattern 01 hp,hp,Tn2,*tnpR*,*bla*$_{\text{TEM-1B}}$,hp,hp,*hns*,hp,hp,hp,hp,hp,hp (n=240)

Pattern 02 hp,hp,Tn2,*tnpR*,*bla*$_{\text{TEM-1B}}$,ISVsa3,hp,*sul2*,hp,IS5075,hp (n=160)

Pattern 03 ISAs25,ISEc63,*tnpR*,*bla*$_{\text{TEM-1B}}$,*tetR*,*tet*(A),hp,hp,hp,hp (n=47)

Pattern 04 Q9RNC7,hp,Tn2,*tnpR*,*bla*$_{\text{TEM-1B}}$,*tetR*,*tet*(A),hp,hp,hp,hp (n=20)*

Pattern 05 hp,*bla*$_{\text{CTX-M-15}}$,ISEcp1,hp,*tnpR*,*bla*$_{\text{TEM-1B}}$,hp,*aph(6)-Id*,*aph(3'')-Ib*,*sul2*,hp,IS5075 (n=11)

Pattern 07 hp,*relB*,*relE*,ISEc63,*tnpR*,*bla*$_{\text{TEM-1B}}$,hp,*aph(6)-Id*,*aph(3'')-Ib*,*sul2*,hp,IS5075 (n=75)

Supplementary figure 7. Dominant flanking region patterns of *Salmonella* Typhi harboring *bla*$_{\text{TEM}}$ isolates. The flanking region patterns were analyzed with 5,000 bp flanking region and others as default setting. The distribution showed six dominant flanking region patterns with gene orientation, 01, 02, 03, 04, 05 and 07 (n=553/577). The grey color is hypothetical genes used for pattern classification. The symbol * denotes a pattern containing *bla*$_{\text{TEM}}$ variant, including *bla*$_{\text{TEM-1B}}$ (n=17), *bla*$_{\text{TEM-135}}$ (n=2) and *bla*$_{\text{TEM-215}}$ (n=1).

Supplementary table 8. Flanking region patterns of *Salmonella* Typhi harboring bla_{TEM} isolates ranked by countries. The flanking region patterns were analyzed with 5,000 bp flanking region and others as default setting.

bla $_{\mathbf{TEM}}$ **isolate by flanking region pattern**	**Total isolate**	**Top 5 country (No of isolate)**
Pattern 01	240	Nigeria (87), Ghana (81), Benin (5), UK (4), France (4), Others (15), Unknown (44)
Pattern 02	160	Bangladesh (142), India (5), USA (3), Canada (3), UK (2), Others (3), Unknown (2)
Pattern 03	47	Nigeria (20), USA (2), UK (2), South Africa (1), Unknown (22)
Pattern 04.1	19	Cameroon (14), Malawi (2), Benin (1), Ghana (1), Unknown (1)
Pattern 04.2	1	Nigeria (1)
Pattern 05	11	UK (4), Pakistan (4), India (3)
Pattern 07	75	Zambia (47), Tanzania (18), South Africa (4), USA (2), Zimbabwe (1), UK (1), Unknown (2)
Pattern 08	2	Philippines (2)
Pattern 09.1	3	China (3)
Pattern 09.2	3	China (3)
Pattern 10	2	Bangladesh (2)
Pattern 11	1	UK (1)
Pattern 13 Others	13	Pakistan (4), UK (2), India (2), Viet Nam (1), Singapore (1), Colombia (1), Bangladesh (1), Indonesia (1)
Pattern 14 No output	5502	Pakistan (1185), Bangladesh (692), Malawi (554), South Africa (537), UK (469), Others (1856), Unknown (209)

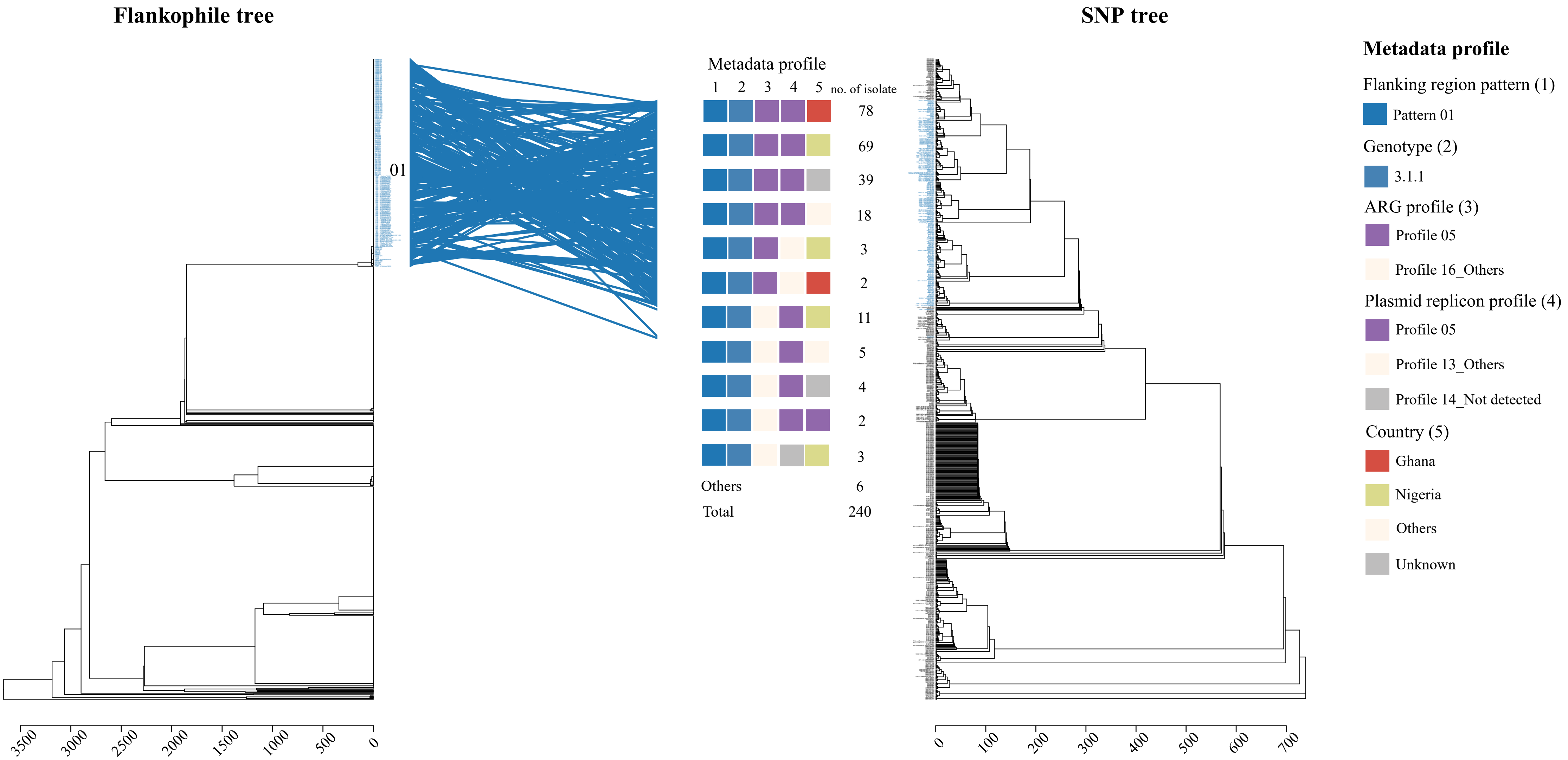

Supplementary figure 8. Clustering of flanking region pattern 01 with metadata profile. The flanking region pattern 01 (1) of *S*. Typhi harboring *bla*$_{\text{TEM}}$ isolates from flankophile output (n=240) is linked to genotype (2), ARG profile (3), plasmid replicon profile (4) and country (5), respectively.

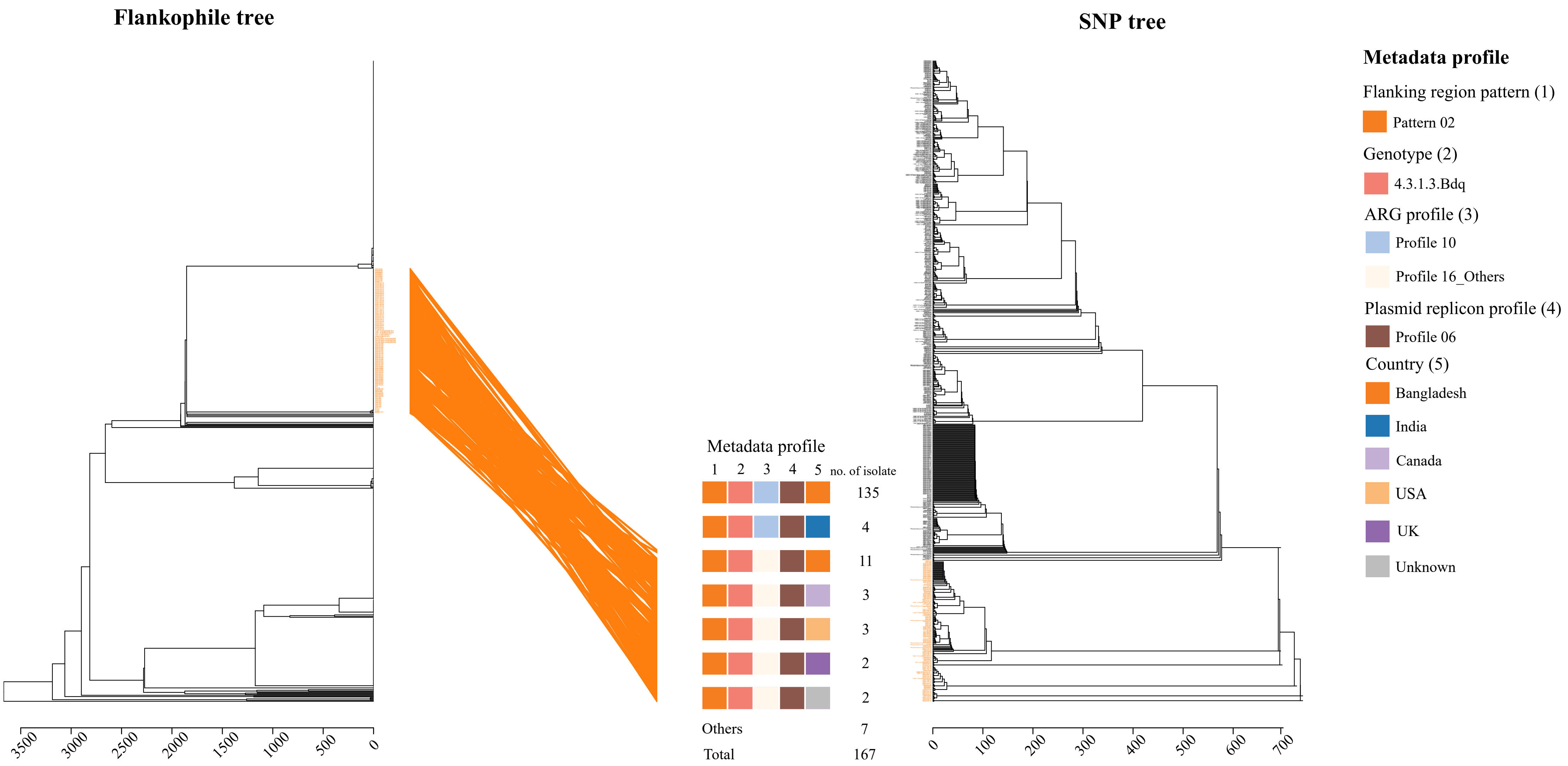

Supplementary figure 9. Clustering of flanking region pattern 02 with metadata profile. The flanking region pattern 02 (1) of *S.* Typhi harboring *bla*$_{\text{TEM}}$ isolates from flankophile output (n=167) is linked to genotype (2), ARG profile (3), plasmid replicon profile (4) and country (5), respectively.

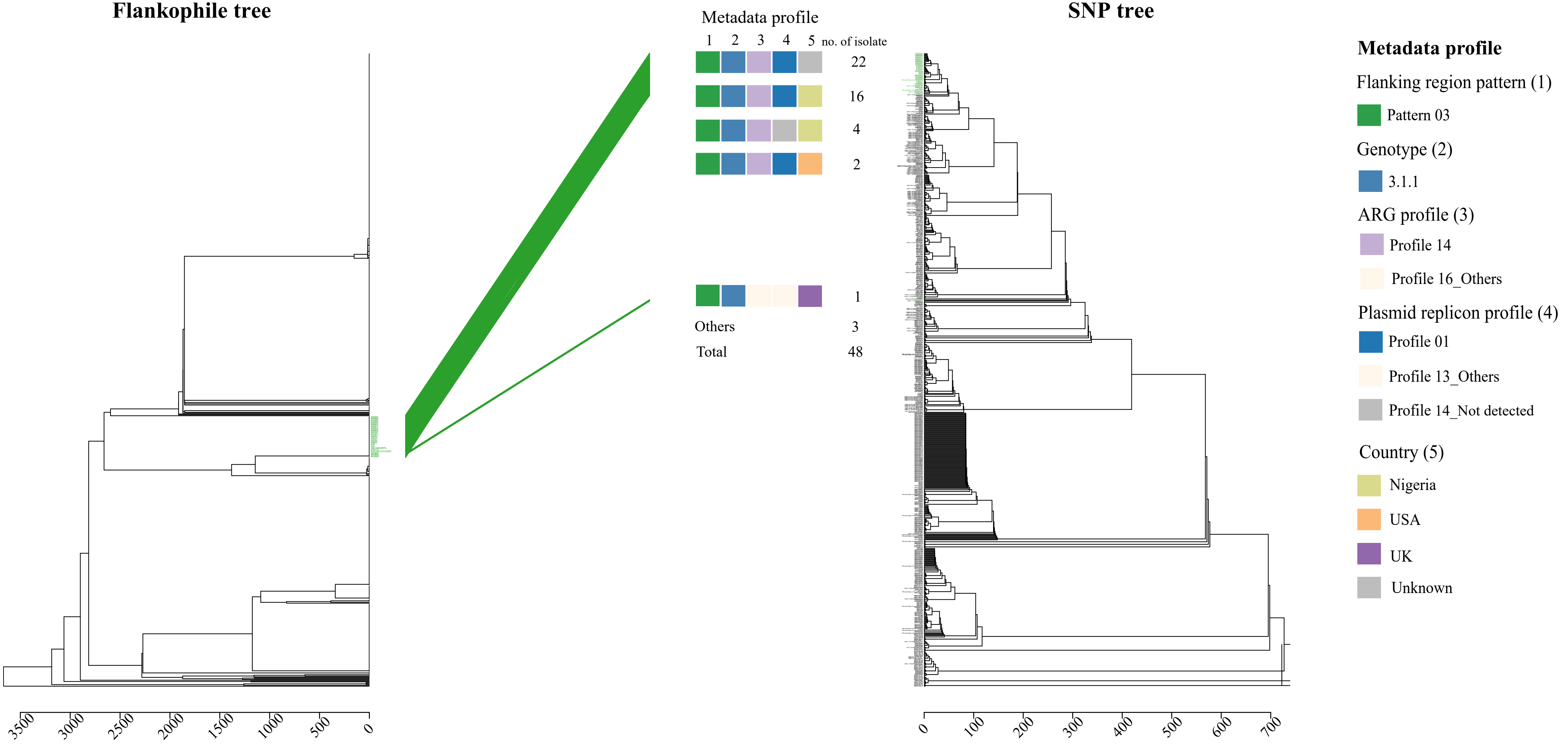

Supplementary figure 10. Clustering of flanking region pattern 03 with metadata profile. The flanking region pattern 03 (1) of *S*. Typhi harboring bla_{TEM} isolates from flankophile output (n=48) is linked to genotype (2), ARG profile (3), plasmid replicon profile (4) and country (5), respectively.

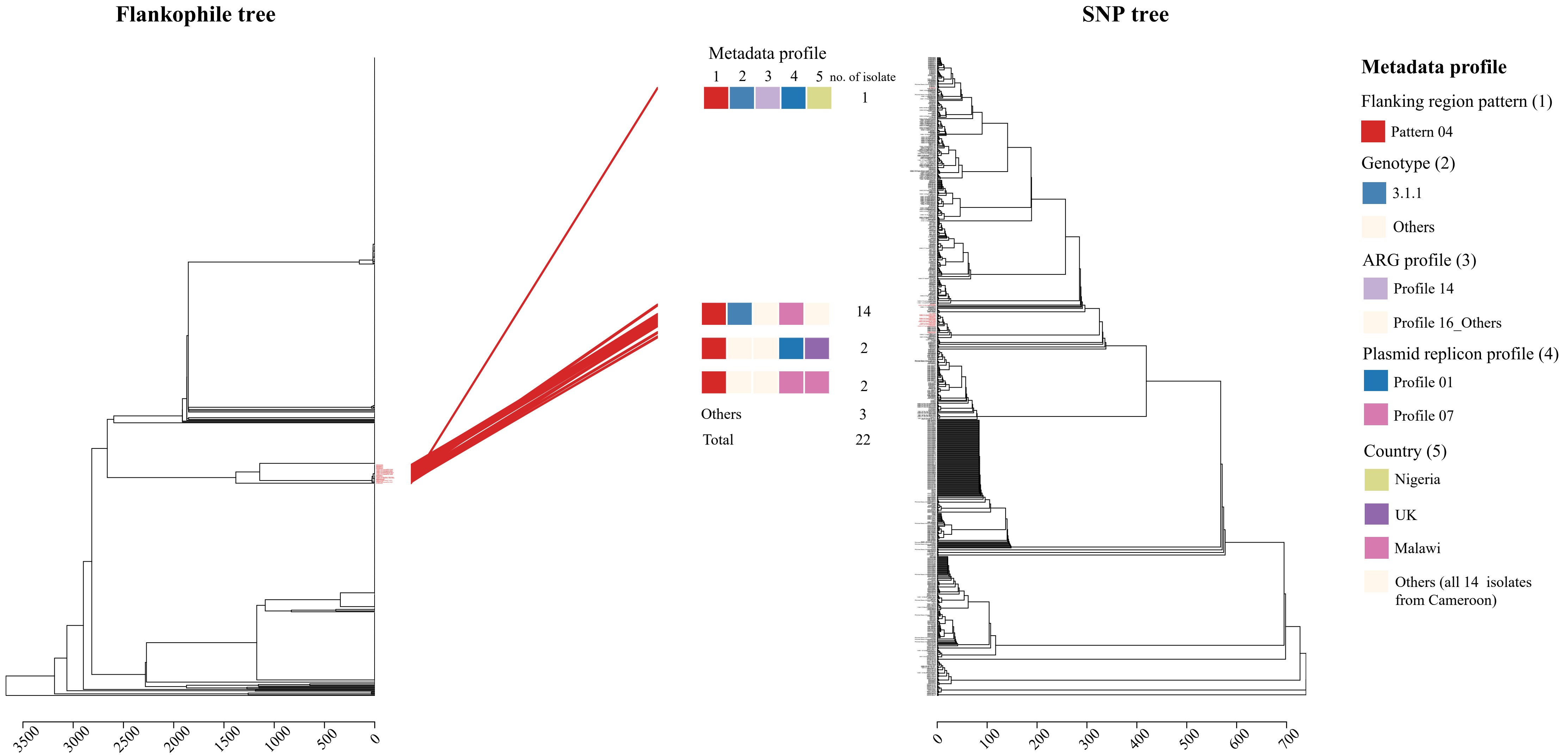

Supplementary figure 11. Clustering of flanking region pattern 04 with metadata profile. The flanking region pattern 04 (1) of *S*. Typhi harboring bla_{TEM} isolates from flankophile output (n=22) is linked to genotype (2), ARG profile (3), plasmid replicon profile (4) and country (5), respectively.

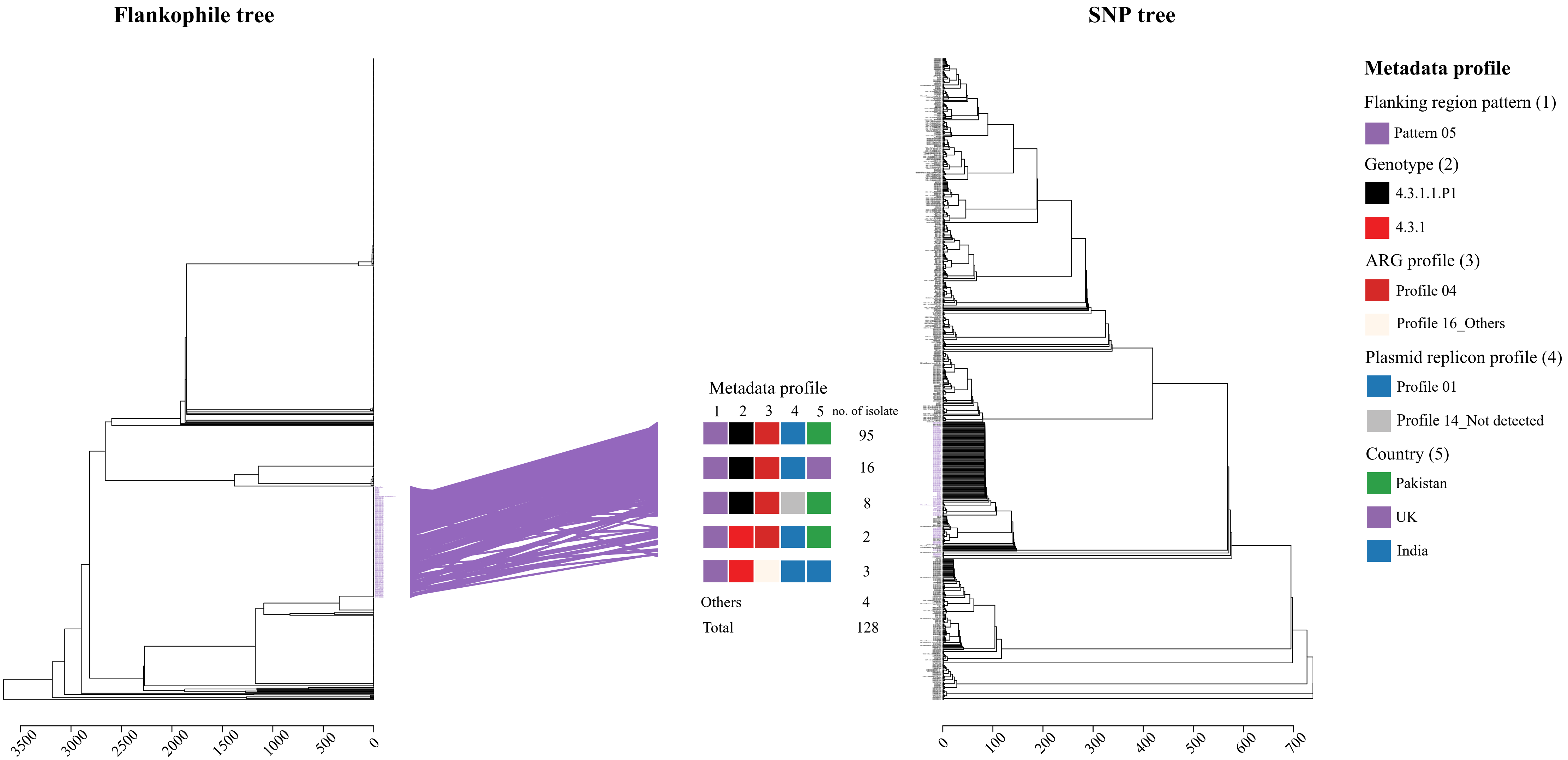

Supplementary figure 12. Clustering of flanking region pattern 05 with metadata profile. The flanking region pattern 05 (1) of *S*. Typhi harboring bla_{TEM} isolates from flankophile output (n=128) is linked to genotype (2), ARG profile (3), plasmid replicon profile (4) and country (5), respectively.

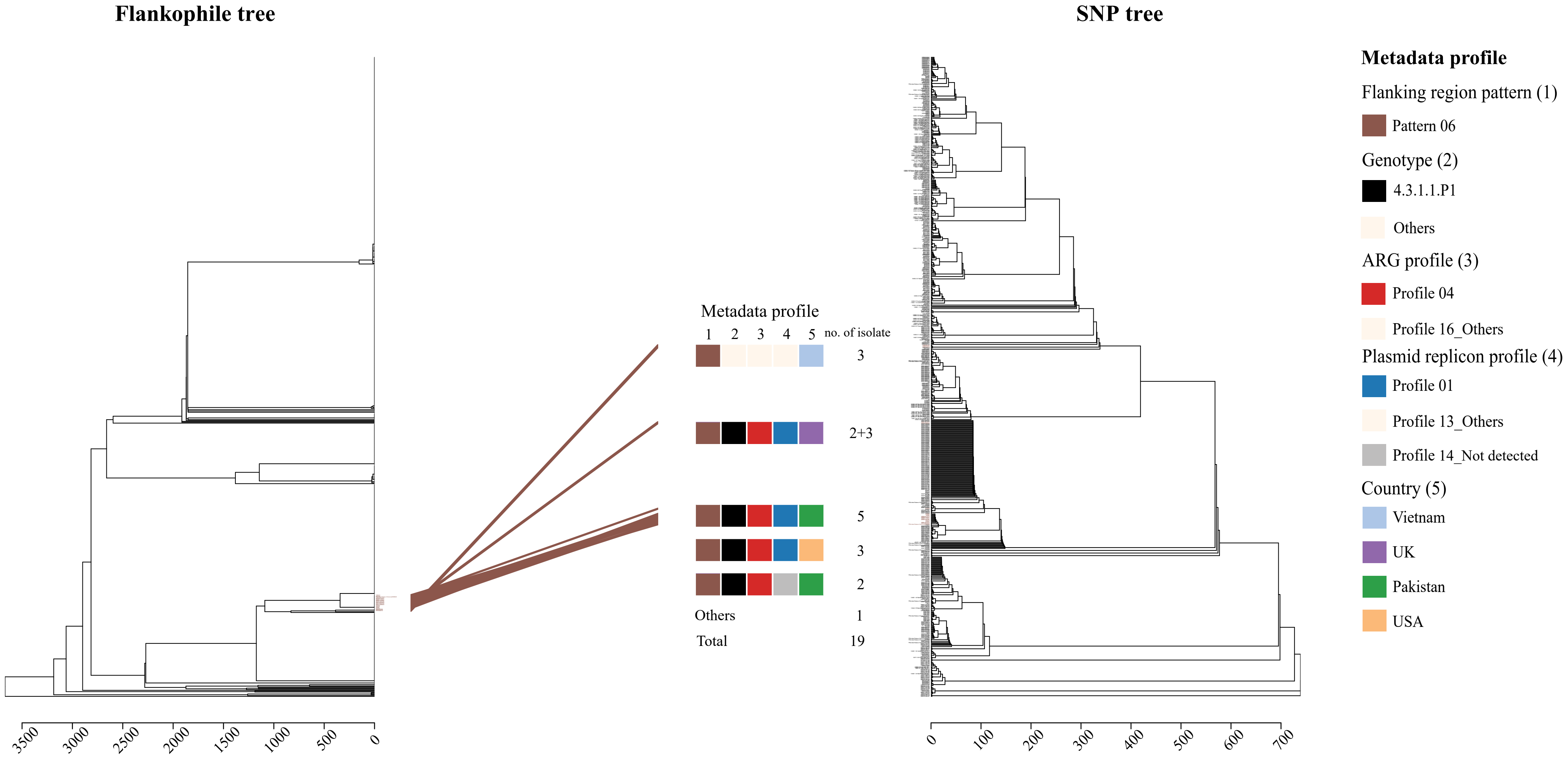

Supplementary figure 13. Clustering of flanking region pattern 06 with metadata profile. The flanking region pattern 06 (1) of *S*. Typhi harboring bla_{TEM} isolates from flankophile output (n=19) is linked to genotype (2), ARG profile (3), plasmid replicon profile (4) and country (5), respectively.

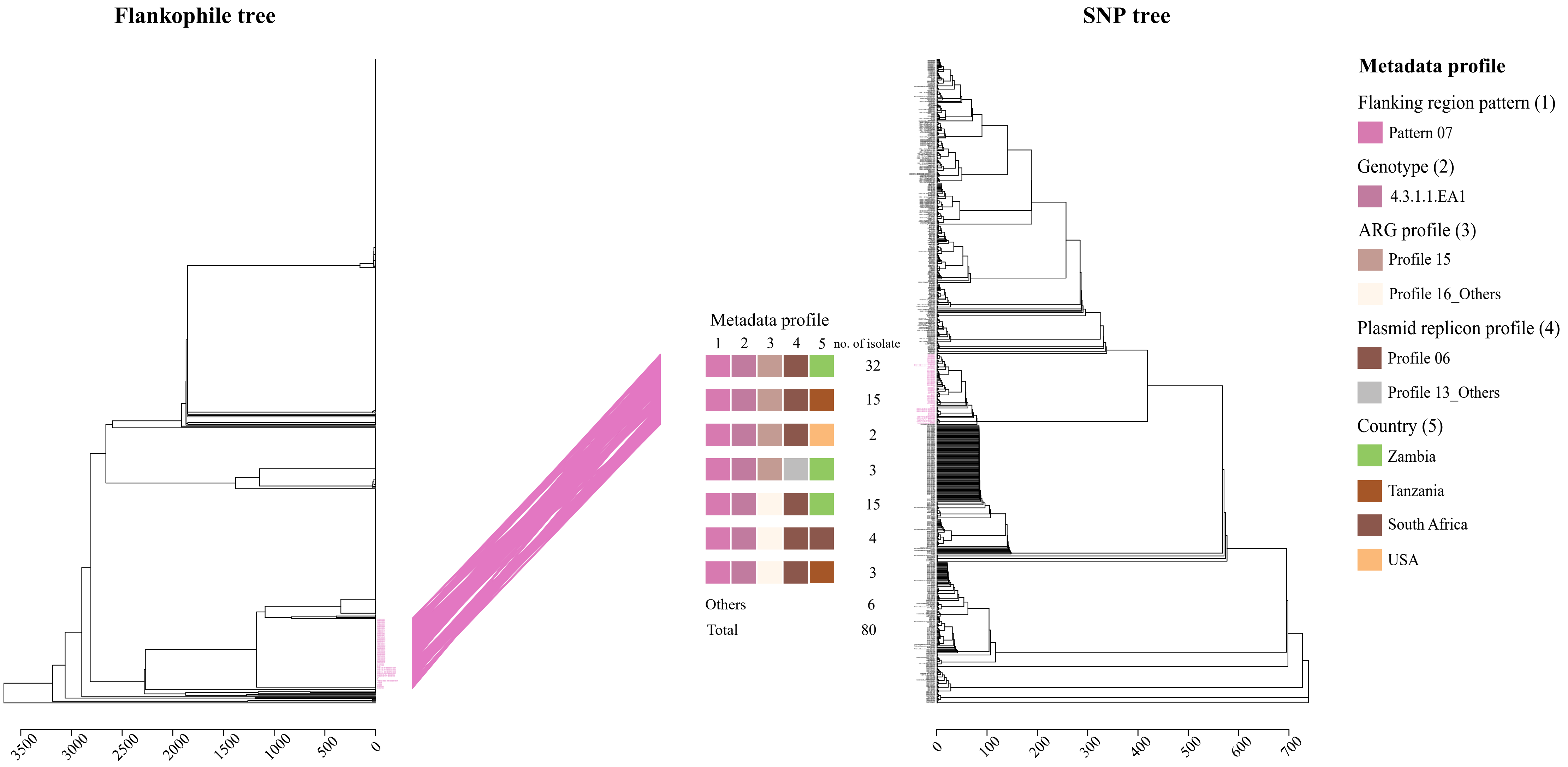

Supplementary figure 14. Clustering of flanking region pattern 07 with metadata profile. The flanking region pattern 07 (1) of *S*. Typhi harboring bla_{TEM} isolates from flankophile output (n=80) is linked to genotype (2), ARG profile (3), plasmid replicon profile (4) and country (5), respectively.

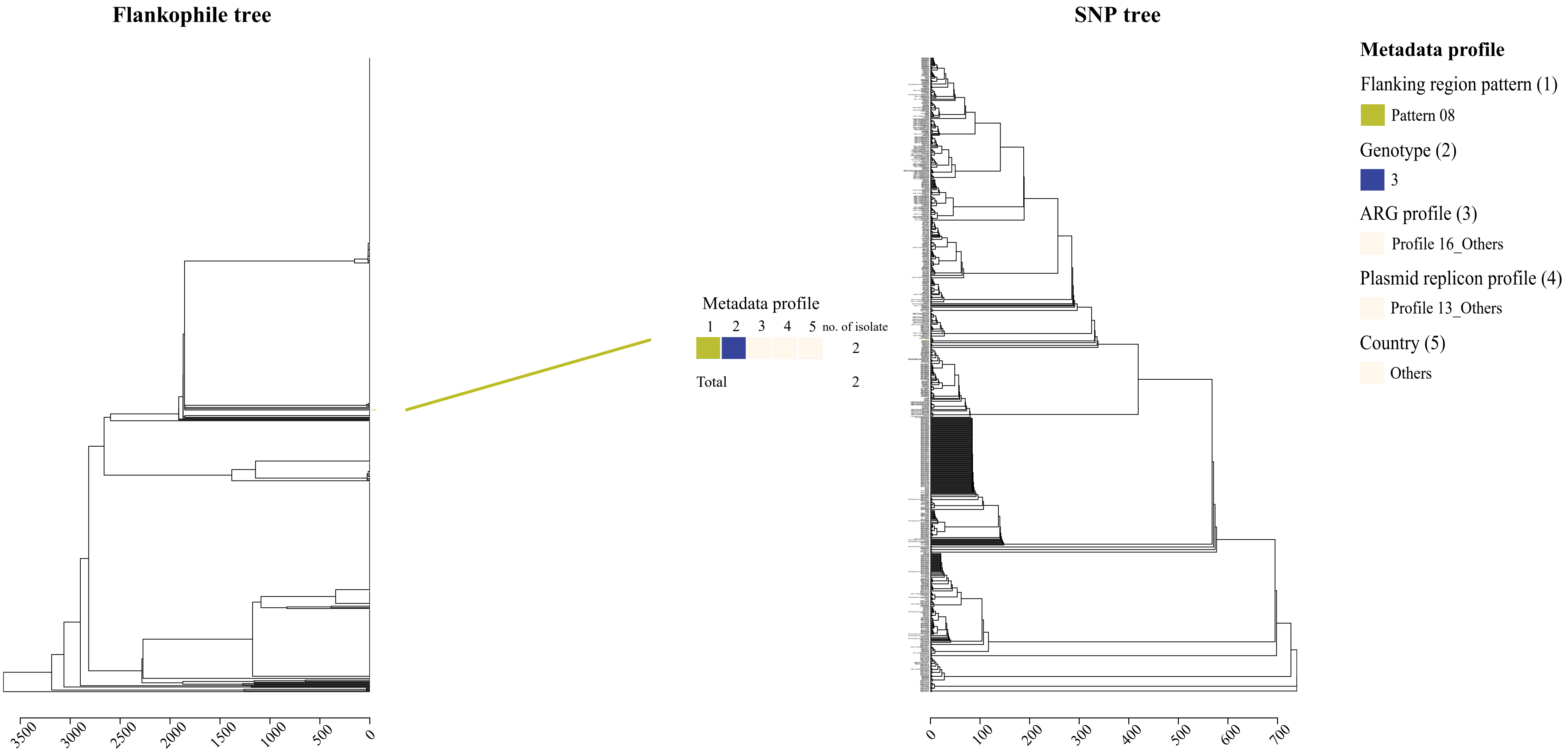

Supplementary figure 15. Clustering of flanking region pattern 08 with metadata profile. The flanking region pattern 08 (1) of *S*. Typhi harboring bla_{TEM} isolates from flankophile output (n=2) is linked to genotype (2), ARG profile (3), plasmid replicon profile (4) and country (5), respectively.

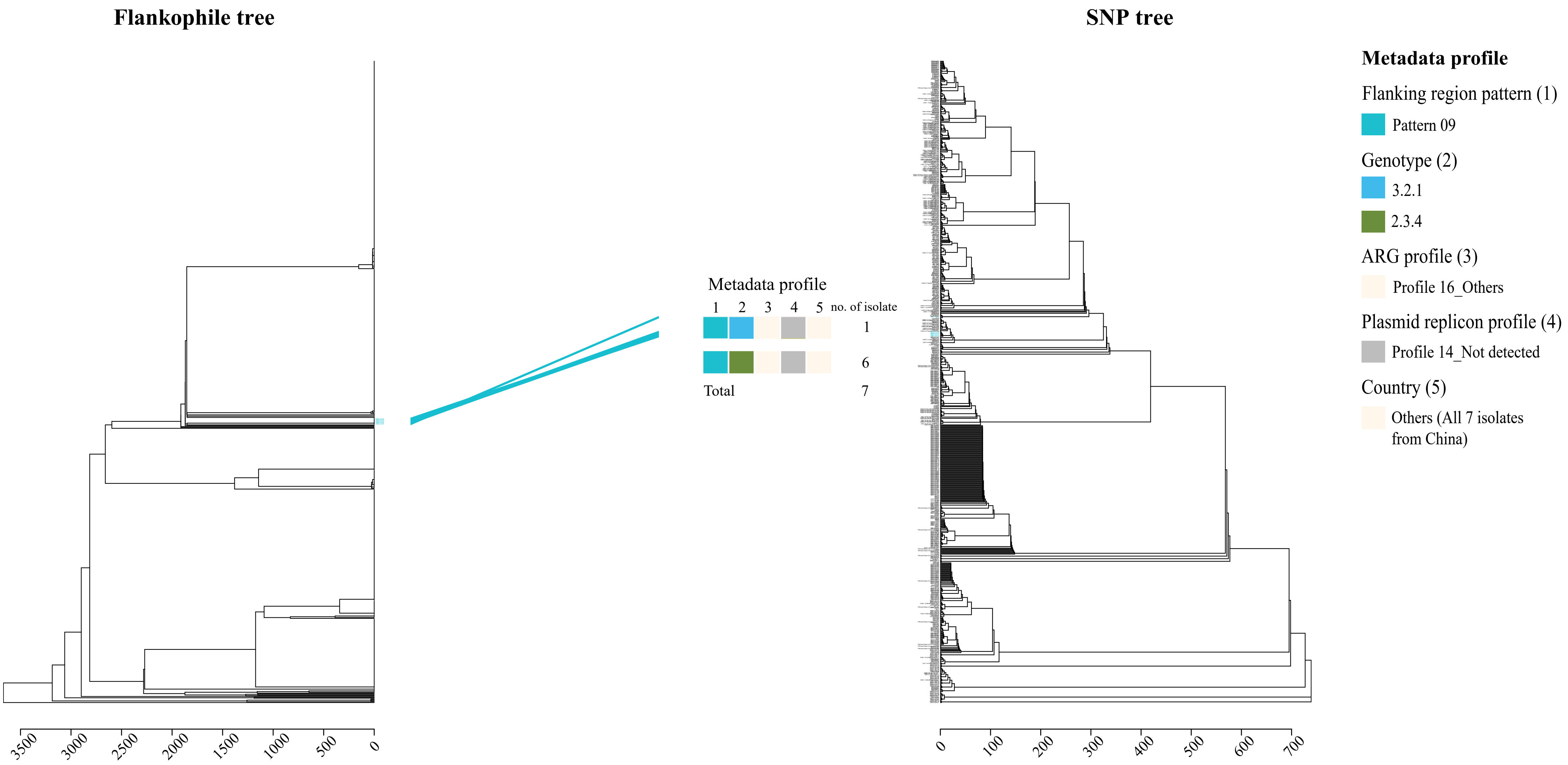

Supplementary figure 16. Clustering of flanking region pattern 09 with metadata profile. The flanking region pattern 09 (1) of *S.* Typhi harboring *bla*$_{\mathrm{TEM}}$ isolates from flankophile output (n=7) is linked to genotype (2), ARG profile (3), plasmid replicon profile (4) and country (5), respectively.

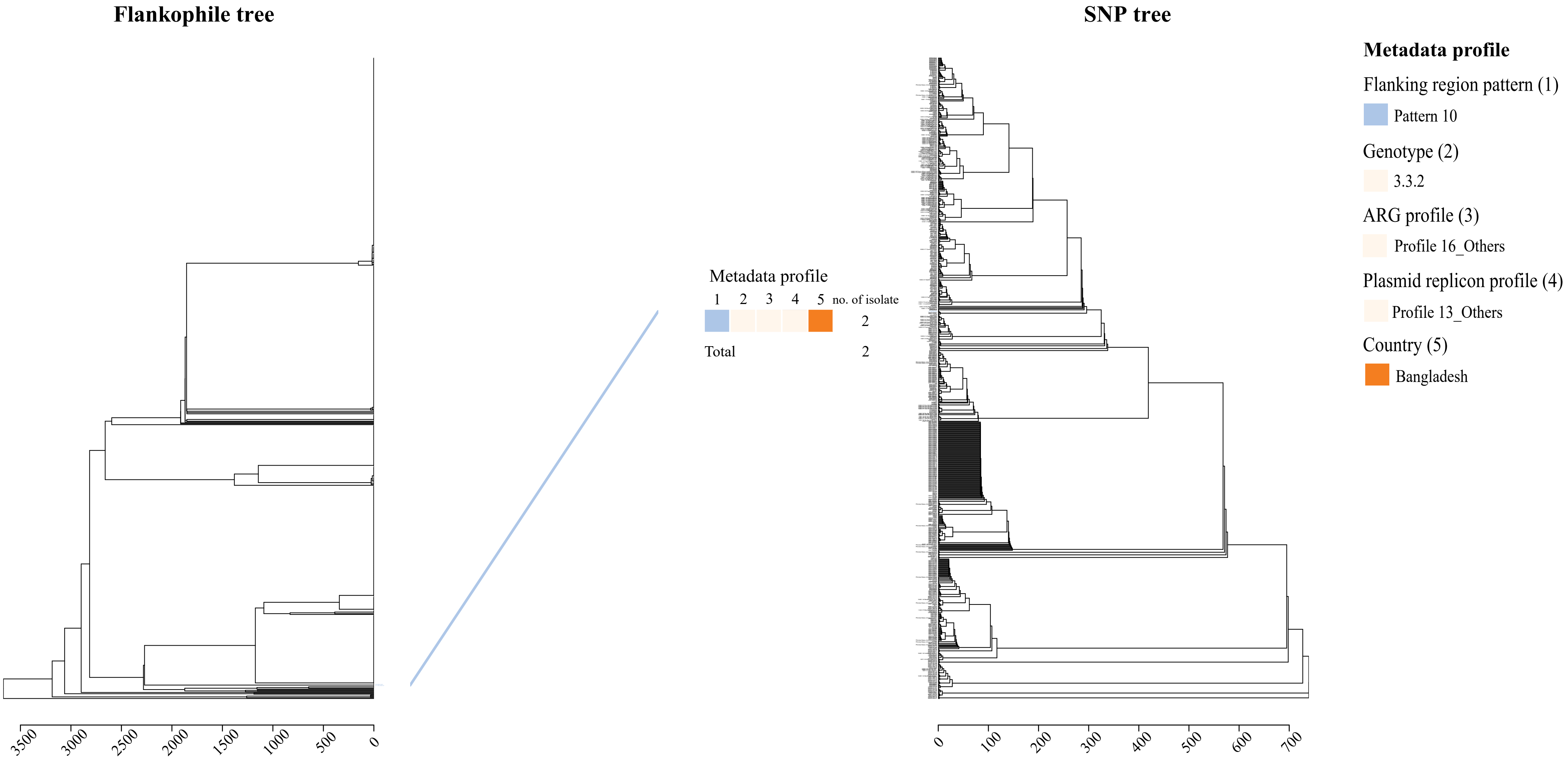

Supplementary figure 17. Clustering of flanking region pattern 10 with metadata profile. The flanking region pattern 10 (1) of *S.* Typhi harboring bla_{TEM} isolates from flankophile output (n=2) is linked to genotype (2), ARG profile (3), plasmid replicon profile (4) and country (5), respectively.

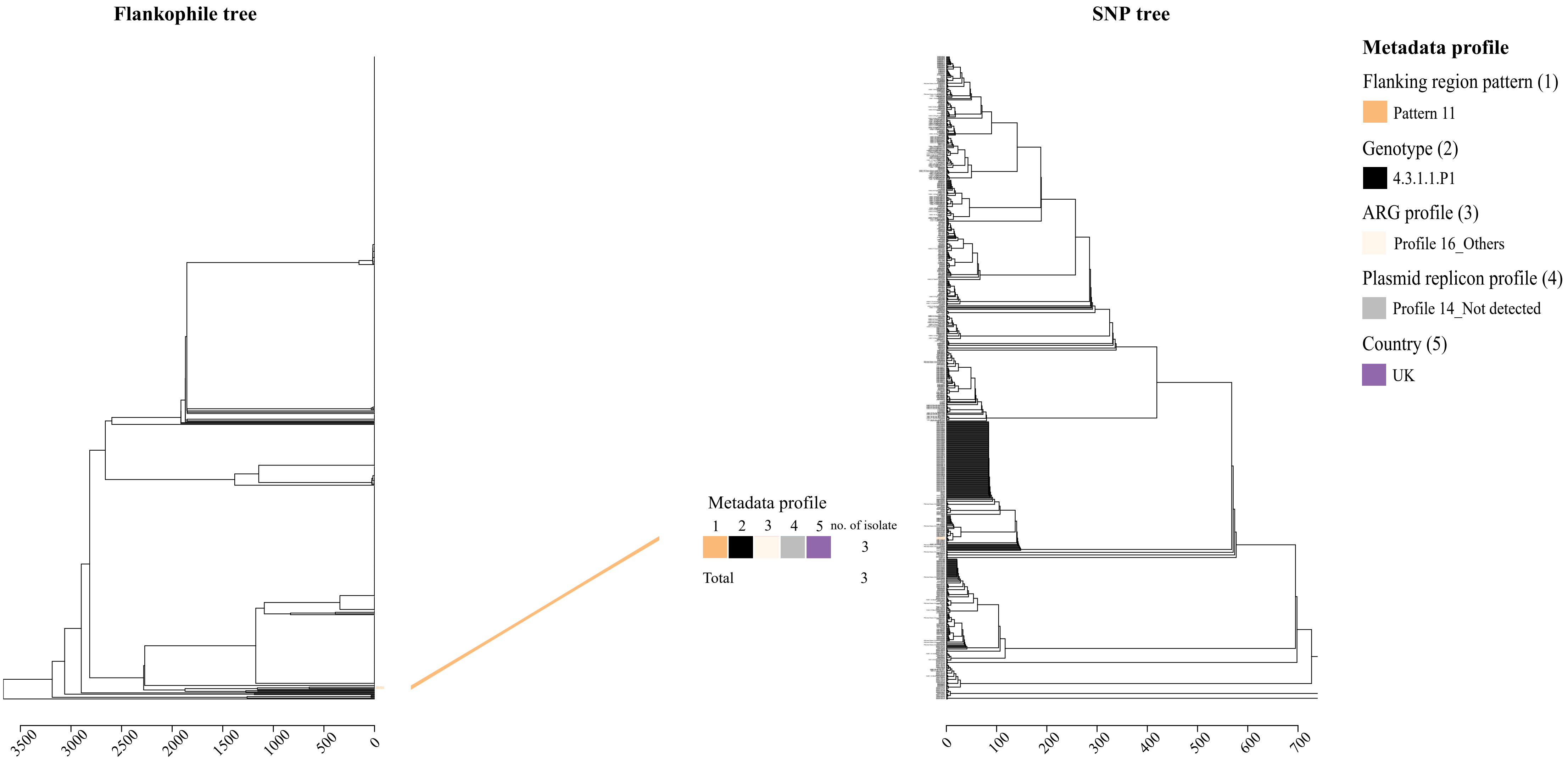

Supplementary figure 18. Clustering of flanking region pattern 11 with metadata profile. The flanking region pattern 11 (1) of *S.* Typhi harboring *bla*$_{\text{TEM}}$ isolates from flankophile output (n=3) is linked to genotype (2), ARG profile (3), plasmid replicon profile (4) and country (5), respectively.

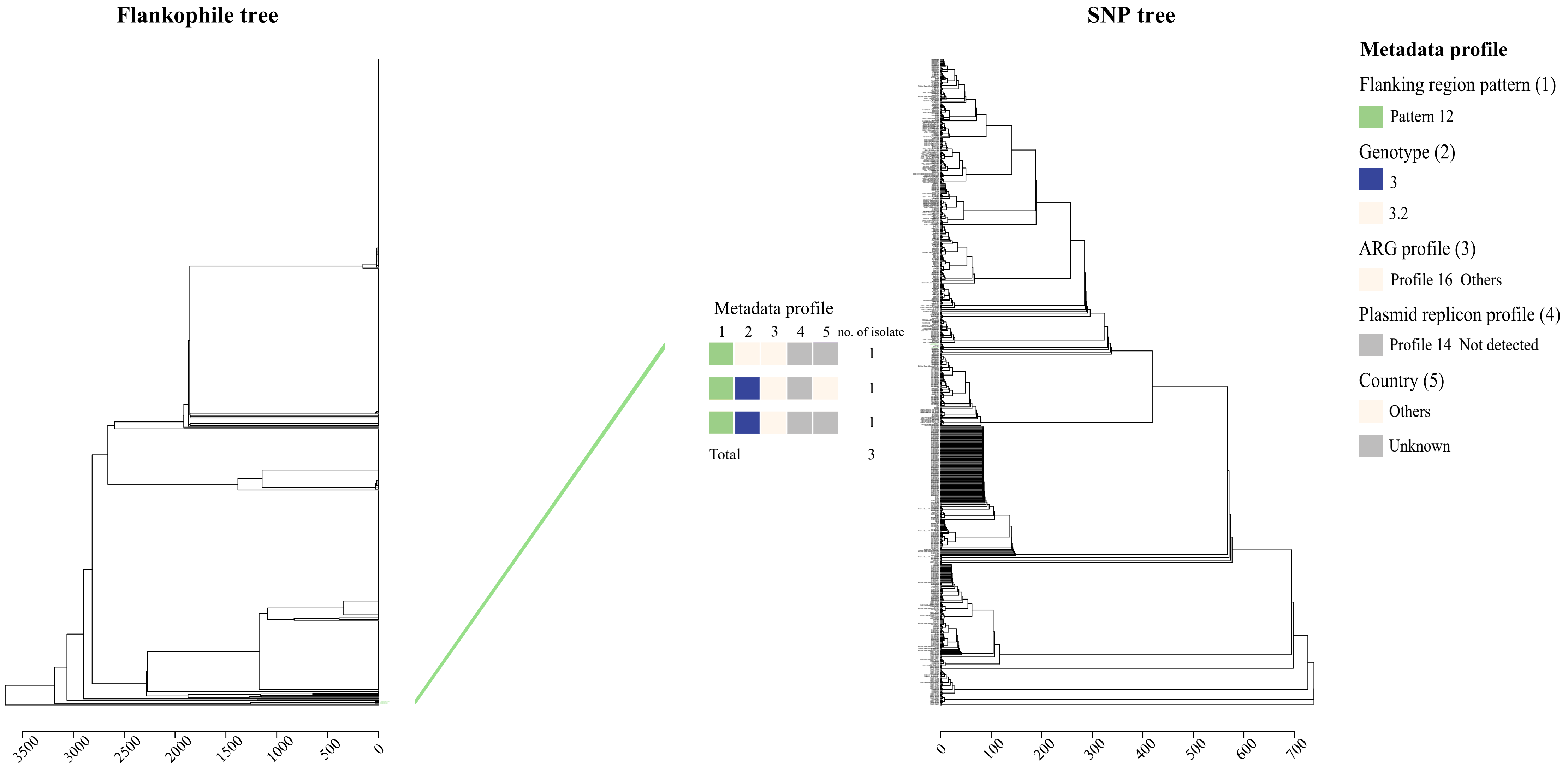

Supplementary figure 19. Clustering of flanking region pattern 12 with metadata profile. The flanking region pattern 12 (1) of *S*. Typhi harboring *bla*$_{\text{TEM}}$ isolates from flankophile output (n=3) is linked to genotype (2), ARG profile (3), plasmid replicon profile (4) and country (5), respectively.

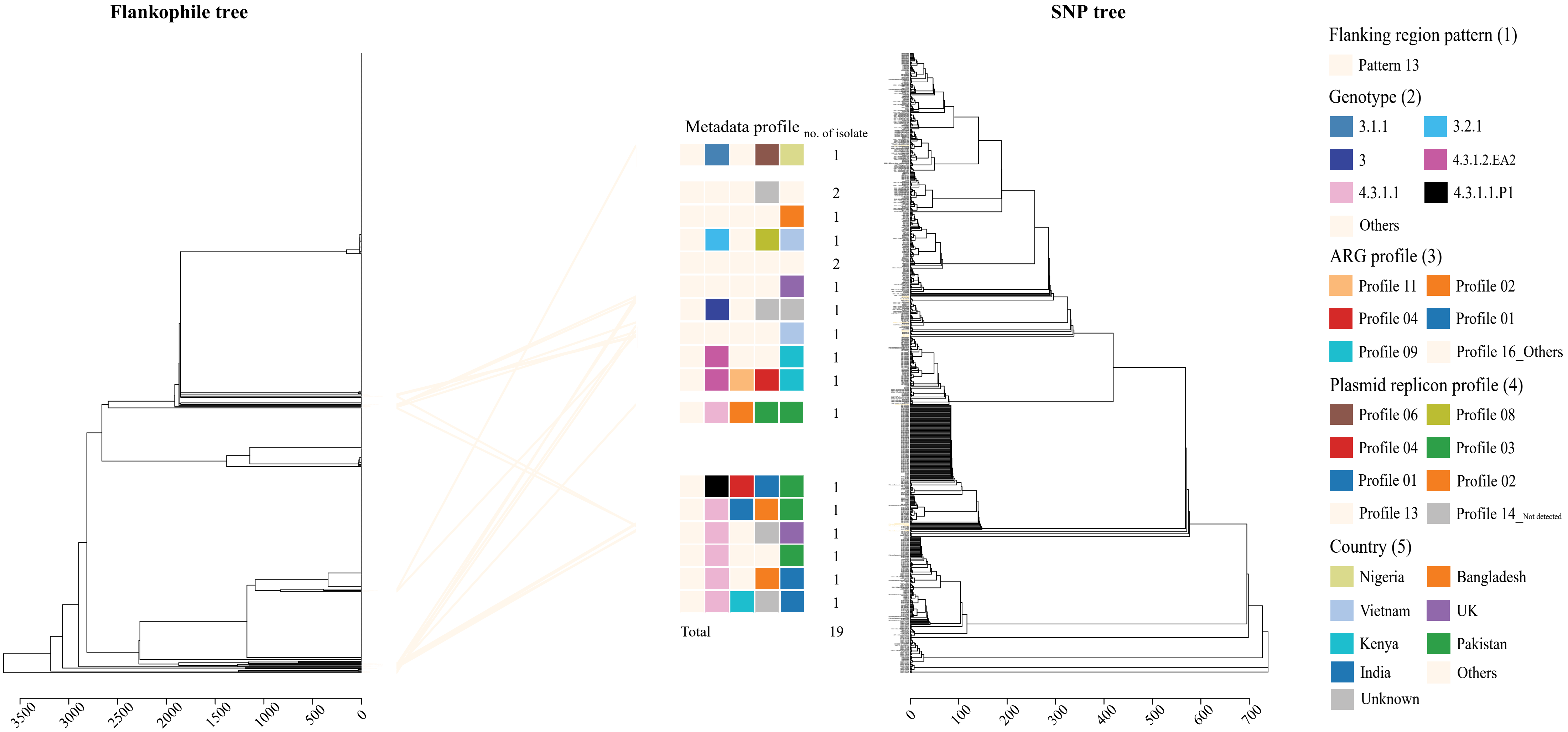

Supplementary figure 20. Clustering of flanking region pattern 13 with metadata profile. The flanking region pattern 13 (1) of *S.* Typhi harboring *bla*$_{\text{TEM}}$ isolates from flankophile output (n=19) is linked to genotype (2), ARG profile (3), plasmid replicon profile (4) and country (5), respectively.
